# Pregnancy-Induced Noncoding RNA (*PINC*) Associates with Polycomb Repressive Complex 2 and Regulates Mammary Epithelial Differentiation

**DOI:** 10.1371/journal.pgen.1002840

**Published:** 2012-07-26

**Authors:** Amy N. Shore, Elena B. Kabotyanski, Kevin Roarty, Martin A. Smith, Yiqun Zhang, Chad J. Creighton, Marcel E. Dinger, Jeffrey M. Rosen

**Affiliations:** 1 Program in Developmental Biology, Baylor College of Medicine, Houston, Texas, United States of America; 2 Department of Molecular and Cellular Biology, Baylor College of Medicine, Houston, Texas, United States of America; 3 Institute for Molecular Bioscience, The University of Queensland, Brisbane, Australia; 4 Dan L. Duncan Cancer Center, Baylor College of Medicine, Houston, Texas, United States of America; 5 Diamantina Institute, The University of Queensland, Princess Alexandra Hospital, Brisbane, Australia; University of Cambridge, United Kingdom

## Abstract

Pregnancy-induced noncoding RNA (*PINC*) and retinoblastoma-associated protein 46 (*RbAp46*) are upregulated in alveolar cells of the mammary gland during pregnancy and persist in alveolar cells that remain in the regressed lobules following involution. The cells that survive involution are thought to function as alveolar progenitor cells that rapidly differentiate into milk-producing cells in subsequent pregnancies, but it is unknown whether *PINC* and RbAp46 are involved in maintaining this progenitor population. Here, we show that, in the post-pubertal mouse mammary gland, *mPINC* is enriched in luminal and alveolar progenitors. *mPINC* levels increase throughout pregnancy and then decline in early lactation, when alveolar cells undergo terminal differentiation. Accordingly, *mPINC* expression is significantly decreased when HC11 mammary epithelial cells are induced to differentiate and produce milk proteins. This reduction in *mPINC* levels may be necessary for lactation, as overexpression of *mPINC* in HC11 cells blocks lactogenic differentiation, while knockdown of *mPINC* enhances differentiation. Finally, we demonstrate that *mPINC* interacts with RbAp46, as well as other members of the polycomb repressive complex 2 (PRC2), and identify potential targets of *mPINC* that are differentially expressed following modulation of *mPINC* expression levels. Taken together, our data suggest that *mPINC* inhibits terminal differentiation of alveolar cells during pregnancy to prevent abundant milk production and secretion until parturition. Additionally, a PRC2 complex that includes *mPINC* and RbAp46 may confer epigenetic modifications that maintain a population of mammary epithelial cells committed to the alveolar fate in the involuted gland.

## Author Summary

During pregnancy, epithelial cells of the mammary gland begin to undergo differentiation into functional alveolar cells that, during lactation, will produce and secrete milk proteins, thereby providing nourishment to offspring. Following lactation, the majority of alveolar cells die and the mammary gland remodels to a pre-pregnancy-like state in a process called involution. However, some alveolar cells survive involution, and these cells are thought to serve as alveolar progenitors that are able to rapidly proliferate and differentiate into milk-producing cells in subsequent pregnancies. Keeping alveolar cells from undergoing terminal differentiation during pregnancy and involution is vital for the preservation of an alveolar progenitor population. Here, we show that the long noncoding RNA, *PINC*, is downregulated in the mammary gland between late pregnancy and early lactation, when alveolar cells begin to terminally differentiate. This reduction of *PINC* levels may be necessary for lactation, as overexpression of *PINC* inhibits differentiation, while knockdown of *PINC* enhances differentiation of mammary epithelial cells. Finally, we find that *PINC* interacts with the chromatin-modifying complex PRC2, suggesting epigenetic regulation may be involved in maintaining alveolar progenitors in the pregnant and involuting mammary gland. These results emphasize the potential importance of lncRNA-PRC2 involvement in regulating cell fate during development.

## Introduction

Noncoding RNAs (ncRNAs) are emerging as significant players in the regulatory circuitry of the cell, rivaling their protein-coding counterparts. Accumulating data have revealed the functional diversity of ncRNAs, in particular long noncoding RNAs (lncRNAs), and their relevance in regulating development and disease [1]–[6]. Previous efforts to understand the function of noncoding RNAs have predominantly focused on small/short RNAs (<200 nucleotides). However, in the past few years there has been an increased focus on lncRNAs, as large-scale analyses have revealed the abundance of these molecules in more complex organisms [7]–[11]. While initial reports of the pervasive noncoding transcription found in the eukaryotic genome were met with skepticism, experimental evidence has shown that many of these lncRNAs are expressed in tissue- and cell-specific patterns in development, suggestive of their functionality [12]–[14]. In addition, knockdown and overexpression studies have shown that an increasing number of lncRNAs play important roles in regulating a diverse spectrum of processes, including splicing [15], transcription [16], localization [17], and organization of subcellular compartments [18].

As more lncRNAs are discovered, functional categorization based on properties such as expression, localization and mechanism allow us to better predict the roles of newly discovered lncRNAs, and have vastly facilitated our understanding of those already known. One recurring feature of an emerging class of lncRNAs is an association with chromatin-modifying complexes. In fact, as many as 38% of large intergenic noncoding RNAs (lincRNAs) have been shown to interact with various chromatin-modifying complexes and 24% specifically interact with polycomb repressive complex 2 (PRC2) [19]. In addition, a recent study has identified thousands more lncRNAs that associate with PRC2, many of which do not fall into the lincRNA category [20]. PRC2 recruitment results in trimethylation of histone H3 at lysine 27 (H3K27), leading to chromatin compaction and transcriptional repression of associated genes. The role of PRC2 in the epigenetic regulation of cohorts of genes involved in the maintenance of cellular identity and differentiation during tissue development is well documented [21]. While the functional importance of the abundant association of lncRNAs with these complexes has, in most cases, not yet been validated, several lncRNAs have been shown to be necessary for targeting PRC2 to specific loci either in *cis* or in *trans*, thereby altering gene expression [13], [22]–[24].

Post-pubertal mammary gland development involves hormonally-regulated expansion and regression of the mammary epithelium as the gland undergoes the processes of pregnancy, lactation and involution [25]–[28]. During pregnancy, mammary epithelial cells (MECs) proliferate and begin to differentiate into highly specialized alveolar cells through a process known as alveologenesis. Terminal differentiation is delayed until parturition, when alveolar cells gain the capacity to produce and secrete the high levels of milk protein necessary for lactation. Following lactation, the majority of differentiated alveolar cells undergo apoptosis and the gland remodels to a pre-pregnancy state in a process called involution. However, a small population of partially committed alveolar cells remains in the involuted gland and functions as alveolar progenitor cells that rapidly differentiate into milk-producing cells in subsequent pregnancies [29]. Although several signaling pathways are known to play a role in the stepwise progression of alveologenesis [30]–[32], the molecular mechanisms that maintain alveolar progenitors in the pregnant and involuting gland are unknown. The maintenance of stem and progenitor populations has been proposed to require epigenetic modifications to stably suppress differentiation, a function well suited for a histone-modifying complex such as PRC2 [33], [34].

Previously, our laboratory identified *P*regnancy-*I*nduced *N*on*C*oding RNA (*PINC*) and retinoblastoma-associated protein 46 (*RbAp46*) as genes that are elevated in the involuted rat mammary gland compared to an age-matched virgin gland [35]. *PINC* and *RbAp46*, a member of PRC2, were shown to be highly expressed in alveolar cells of the pregnant gland and in MECs that remain in the regressed lobules following involution. *PINC* is a mammalian-specific, evolutionary conserved, alternatively spliced and polyadenylated lncRNA. Initial *in vitro* studies of mouse *PINC* (*mPINC*) splice forms, *mPINC1.0* and *mPINC1.6*, showed potential roles in the regulation of survival and cell cycle progression of mammary epithelial cells [36]. However, the developmental regulation of *PINC* and *RbAp46* in the post-pubertal mammary gland, as well as their cell-type specificity, suggest they may play a more specialized role in alveologenesis. Therefore, the objective of this study was to evaluate a role for *mPINC* in regulating alveolar development and investigate a potential interaction of *mPINC* and RbAp46.

In the present study, we show that *mPINC* expression declines in the mammary gland during the transition from late pregnancy to early lactation as well as in response to lactogenic hormone-induced differentiation of the HC11 mammary epithelial cell line. We also show that overexpression of *mPINC* blocks lactogenic differentiation, while knockdown of *mPINC* enhances lactogenic differentiation of HC11 cells. In addition, we provide evidence for an interaction between *mPINC* and RbAp46, as well as other members of PRC2. Finally, we identify potential *mPINC* targets that are differentially expressed following modulation of *mPINC* levels. Taken together, these data suggest that *mPINC* inhibits differentiation of alveolar cells and that *mPINC* downregulation is necessary during lactation to allow alveolar cells to undergo terminal secretory differentiation. Additionally, a PRC2 complex that includes *mPINC* and RbAp46 may provide the epigenetic modifications that maintain a progenitor population of MECs committed to an alveolar fate in the pregnant and involuted gland.

## Results

### *mPINC* is alternatively spliced and differentially regulated in the post-pubertal mouse mammary gland

**Figure 1:**
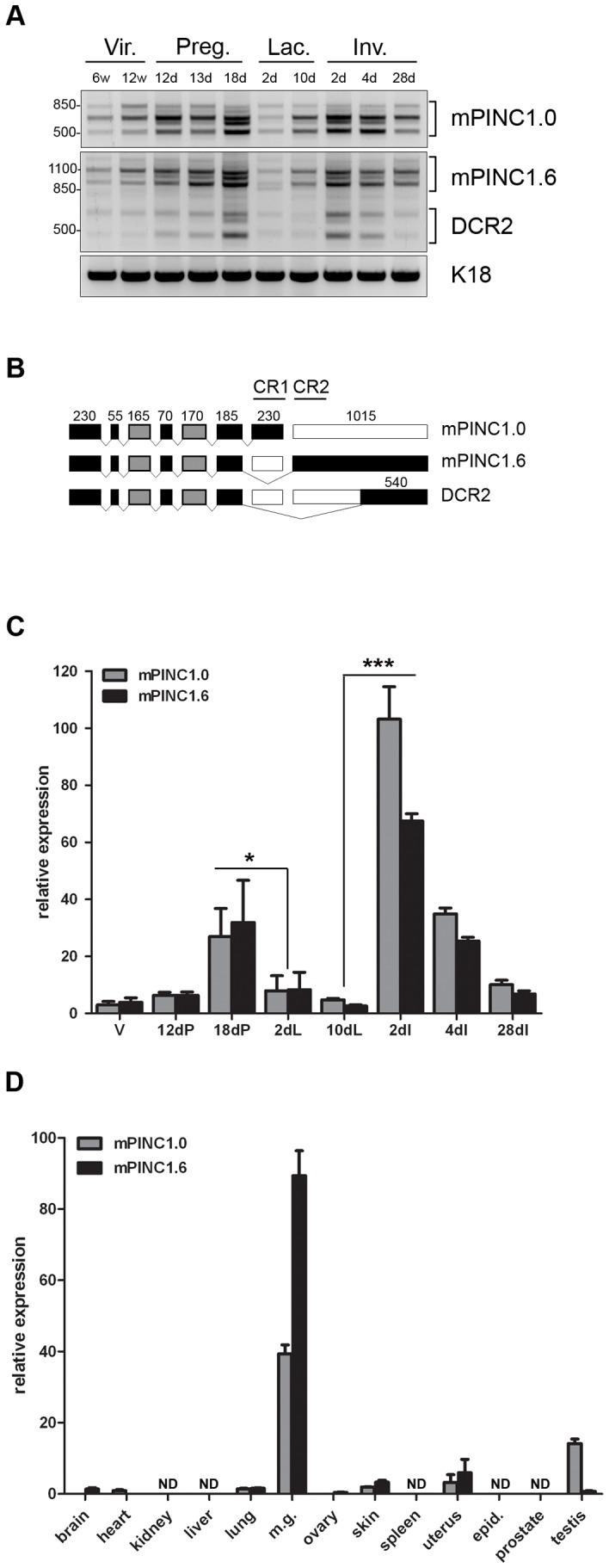
*mPINC* expression peaks in the late pregnant and early involuting gland. (A) RT-PCR shows multiple splice forms of *mPINC1.0* and *mPINC1.6* are expressed during mammary gland development. Primers designed to the extreme ends of *mPINC1.0* and *mPINC1.6* were used to amplify cDNA from mammary gland developmental stages. (w Vir.: weeks old virgin, d Preg.: days pregnancy, d Lac.: days lactation, d Inv.: days involution). PCR products were sequenced and found to be new splice forms of *mPINC*, including a new splice variant of *mPINC1.6* called *DCR2*, for deleted conserved region 2. (B) Schematic diagram of *mPINC* exonic structure. Black boxes represent exons that are always included, grey boxes are sometimes included and clear boxes are never included. Nucleotide length is indicated above each exon along with black lines that overlap the most conserved regions of the *PINC* locus, CR1 and CR2. Exon 6 sometimes has an additional 24 nucleotides at the 3′ end in the *mPINC1.0* and *mPINC1.6* splice forms. This alternative splice site does not correlate with the inclusion/exclusion of any particular exon and its function is unknown. (C) qPCR shows *mPINC* is highest during late pregnancy and early involution. Mammary glands were harvested from 3 female Balb/c mice for each stage (V: adult virgin, dP: days pregnant, dL: days lactation, dI: days involution). Target genes were normalized to *Actb* and set relative to levels in the virgin mammary gland. (D) *mPINC* expression is most abundant in the mammary gland. Tissues were harvested from three 10 week old virgin Balb/c female mice and testis, epididymis, and prostate was harvested from three 12 week old male Balb/c mice. ND indicates tissues in which *mPINC* was not detected by qPCR. Target genes were normalized to *Actb* and set relative to levels in the lung.

*PINC* was initially isolated from the rat mammary gland. We therefore decided to investigate the localization and expression levels of *PINC* in the mouse. Previously, we identified two *mPINC* splice forms, *mPINC1.0* and *mPINC1.6*, based on available EST data [36]. To analyze expression of *mPINC* during post-pubertal mouse mammary development, RT-PCR was performed using primers at the extreme ends of each splice form. We found that *mPINC* levels were low in the virgin gland, increased during pregnancy, declined in early lactation, and increased again in early involution (Figure 1A). Unexpectedly, *mPINC1.0* and *mPINC1.6* RT-PCR amplified multiple bands, indicating each splice form may be further spliced. Each band was cloned and sequenced, which verified that there are at least three *mPINC1.0* splice forms and six *mPINC1.6* splice forms. Of the six *mPINC1.6* splice forms, three lack the highly conserved region of the unique terminal exon of *mPINC1.6* and were therefore designated *DCR2* (deleted conserved region 2) (Figure 1B).

The RT-PCR data suggest that *mPINC1.0* and *mPINC1.6* levels are coordinately regulated during mammary gland development. We, therefore, performed quantitative PCR (qPCR) to more precisely quantify expression levels of *mPINC* splice forms. Both *mPINC1.0* and *mPINC1.6* increased throughout pregnancy and then declined between day 18 of pregnancy and day 2 of lactation (Figure 1C). Expression increased again at day 2 of involution, declining thereafter until 28 days post-involution, where both splice forms were still elevated compared to the virgin gland. These data are in agreement with previous observations from the rat mammary gland and verify that *mPINC* splice forms are, in general, coordinately regulated during mammary gland development.

*PINC* shows mammalian-specific conservation and previously *PINC* expression was found to be largely restricted to the mammary gland in the rat [36]. As mentioned above, there are only two mouse ESTs related to *PINC* in the GenBank database and they are both from mammary gland libraries. To obtain a more thorough analysis of *PINC* expression in the mouse, qPCR was performed using RNA isolated from a diverse range of tissues. The results showed that *mPINC* was expressed at very low levels in non-mammary tissues compared to virgin mammary gland, including the uterus and testis (Figure 1D). Interestingly, these two tissues undergo hormone-dependent cyclical expansion and regression in the adult, like the mammary gland. However, *mPINC* was greatly enriched in the virgin mammary gland compared to other mouse tissues, and is especially high in the pregnant and involuting mammary gland, suggesting it may have a more specialized role in mammary gland development.

### *mPINC* is upregulated in the luminal cells during pregnancy and is enriched in luminal and alveolar progenitors

**Figure 2:**
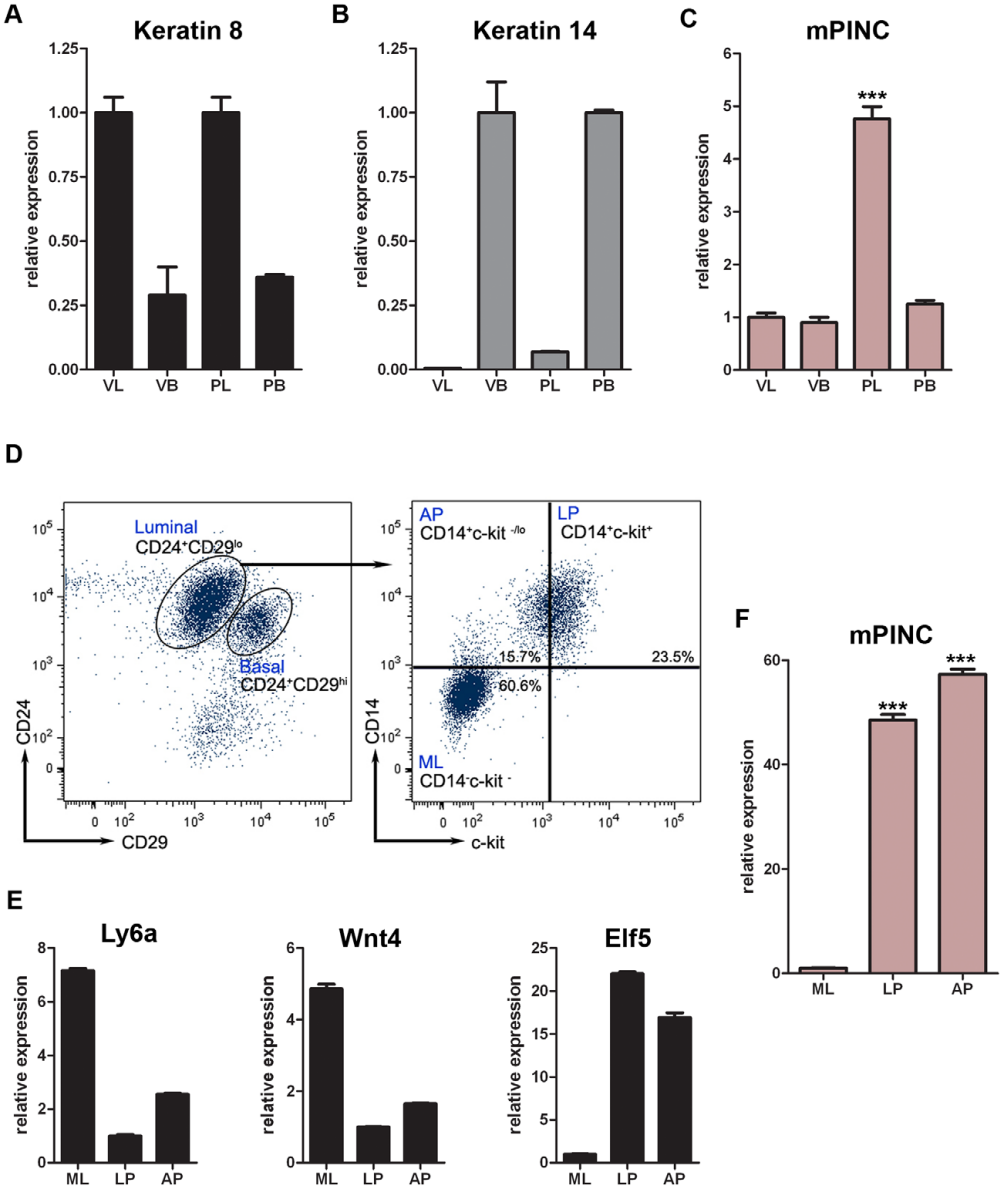
*mPINC* rises specifically in the luminal compartment during pregnancy and is enriched in luminal and alveolar progenitors of the mammary gland. (A–C) RNA was isolated from FACs sorted mammary populations including, virgin luminal (VL) and basal (VB) as well as pregnant luminal (PL) and basal (PB). (A) qPCR showed *Krt8* expression was enriched in the luminal populations (VL and PL), (B) *Krt14* was enriched in the basal populations (VB and PB) and (C) *mPINC* was enriched in the pregnant luminal population, (D–F) MECs were FACs sorted into luminal and basal populations using CD24 and CD29. The luminal population was selected and further sorted into mature luminal (ML), luminal progenitors (LP), and alveolar progenitors (AP) using CD14 and ckit. (D) FACs dot plots showing CD24 and CD29 (left panel) as well as CD14 and ckit (right panel) from virgin MECs. (E) qPCR showed *Ly6a* and *Wnt4* enriched in the ML population and *Elf5* in the LP population thus verifying the purity of each population. (F) *mPINC* was enriched in the luminal progenitors and alveolar progenitors. Data represent mean ±SD (n = 3). Target genes were normalized to *Gapdh*.

*mPINC* is expressed at low levels in the virgin mammary gland and is upregulated throughout pregnancy, however, the cell-type specificity of *mPINC* is unknown. The mammary gland contains two general populations of epithelial cells: basal and luminal [26]. Basal cells include myoepithelial cells, which are contractile and necessary for milk secretion during lactation, as well as a population of stem cells. Luminal cells are comprised of a population of mature, hormone receptor-positive cells and progenitor cells, which are thought to generate ductal and alveolar cells. To determine which epithelial cells of the mouse mammary gland express *mPINC*, we used fluorescence-activated cell sorting (FACS) combined with qPCR. Mammary epithelial cells from 16 day pregnant and age-matched virgin mice were sorted into basal (CD24^lo^CD49f^hi^) and luminal (CD24^hi^CD49f^lo^) cell populations. To confirm the purity of the sorted populations, qPCR was performed and showed elevated levels of keratin 8 (*Krt8*) in the luminal population and keratin 14 (*Krt14*) in the basal population (Figure 2A, 2B). In the virgin mouse, basal and luminal cells expressed equivalent levels of *mPINC*. However, *mPINC* specifically increased in the luminal cells during pregnancy, with expression levels increasing 5-fold compared to the basal cells (Figure 2C). These data are consistent with a role for *mPINC* in regulating alveolar development as the luminal cells are thought to give rise to the alveolar cells during pregnancy.

Next, we wanted to determine if *mPINC* is enriched in a particular subpopulation of luminal mammary epithelial cells. For this purpose, we used mammary epithelial cells from adult virgin mice where three luminal populations have recently been identified and characterized based on expression of CD14 and ckit [37]. These include mature luminal cells (CD14^−^ ckit^−^) and two distinct luminal progenitor populations (CD14^+^ckit^+^ and CD14^+^ckit^−/lo^). The CD14^+^ckit^+^ population resembles the previously described CD24^hi^CD49f^lo^CD61^+^ luminal progenitor population [38], while the CD14^+^ckit^−/lo^ population is thought to be comprised of more committed alveolar progenitor cells. Therefore, we isolated luminal mammary epithelial cells (CD29^lo^CD24^+^) from virgin mice and sorted them into mature luminal cells (CD14^−^ ckit^−^), luminal progenitors (CD14^+^ckit^+^), and alveolar progenitors (CD14^+^ckit^−/lo^) (Figure 2D). To validate the purity of the sorted populations, we used genes that were previously shown to be enriched in either mature luminal or luminal progenitor populations [39]–[41]. qPCR analysis showed increased expression of *Wnt4* and *Ly6a* or *Sca1* (lymphocyte antigen 6 complex locus a) in the mature luminal population as well as *Elf5* (e74-like factor 5) enrichment in the luminal progenitor populations, as expected (Figure 2E). We then analyzed *mPINC* expression and found that it was increased more than 40-fold in the luminal progenitors and 50-fold in the alveolar progenitors compared to the mature luminal population (Figure 2F). The high level of *mPINC* observed in both the luminal and alveolar progenitors supports a potential role for *mPINC* in maintaining mammary epithelial cells in an undifferentiated state.

**Figure 3:**
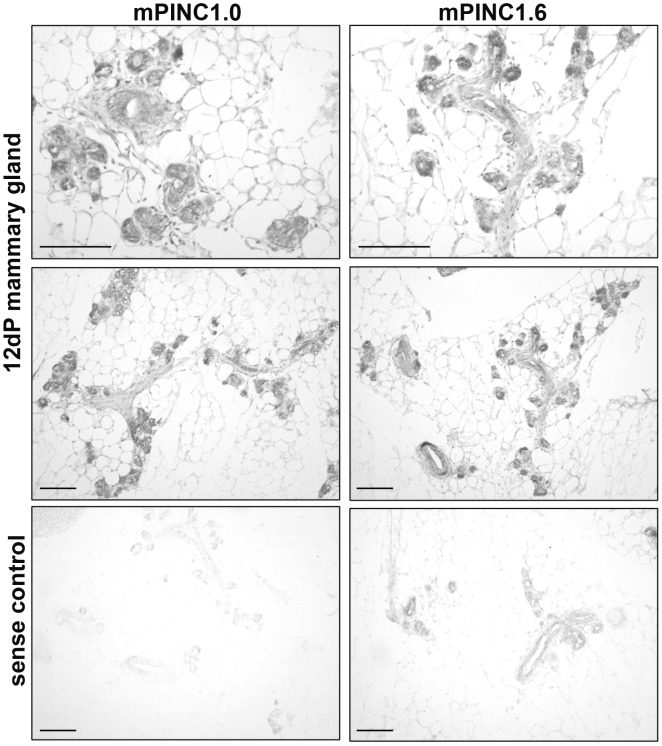
*mPINC* is enriched in alveolar cells of the pregnant gland. *In situ* hybridization using DIG-labeled probes shows *mPINC1.0* and *mPINC1.6* are expressed in alveolar cells of the 12 day mouse mammary gland. Sense control probes for *mPINC1.0* and *mPINC1.6* show very little signal in the bottom panels. Scale bars represent 100 µm.

To verify that *mPINC* is enriched in alveolar cells of the luminal compartment during pregnancy, we performed *in situ* hybridization using probes specific to either *mPINC1.0* or *mPINC1.6* on mammary gland sections from mice at day 12 of pregnancy. These results showed that *mPINC1.0* and *mPINC1.6* were both highly expressed in alveolar cells of the mid-pregnant gland (Figure 3). The majority of alveolar cells expressed *mPINC* in the mid-pregnant gland, although the intensity of the *in situ* signal varied slightly among the alveolar clusters (Figure S1). These localization studies provide further evidence that *mPINC* may play a role in alveolar development.

### *mPINC* is downregulated during lactogenic hormone-induced differentiation of HC11 cells

**Figure 4:**
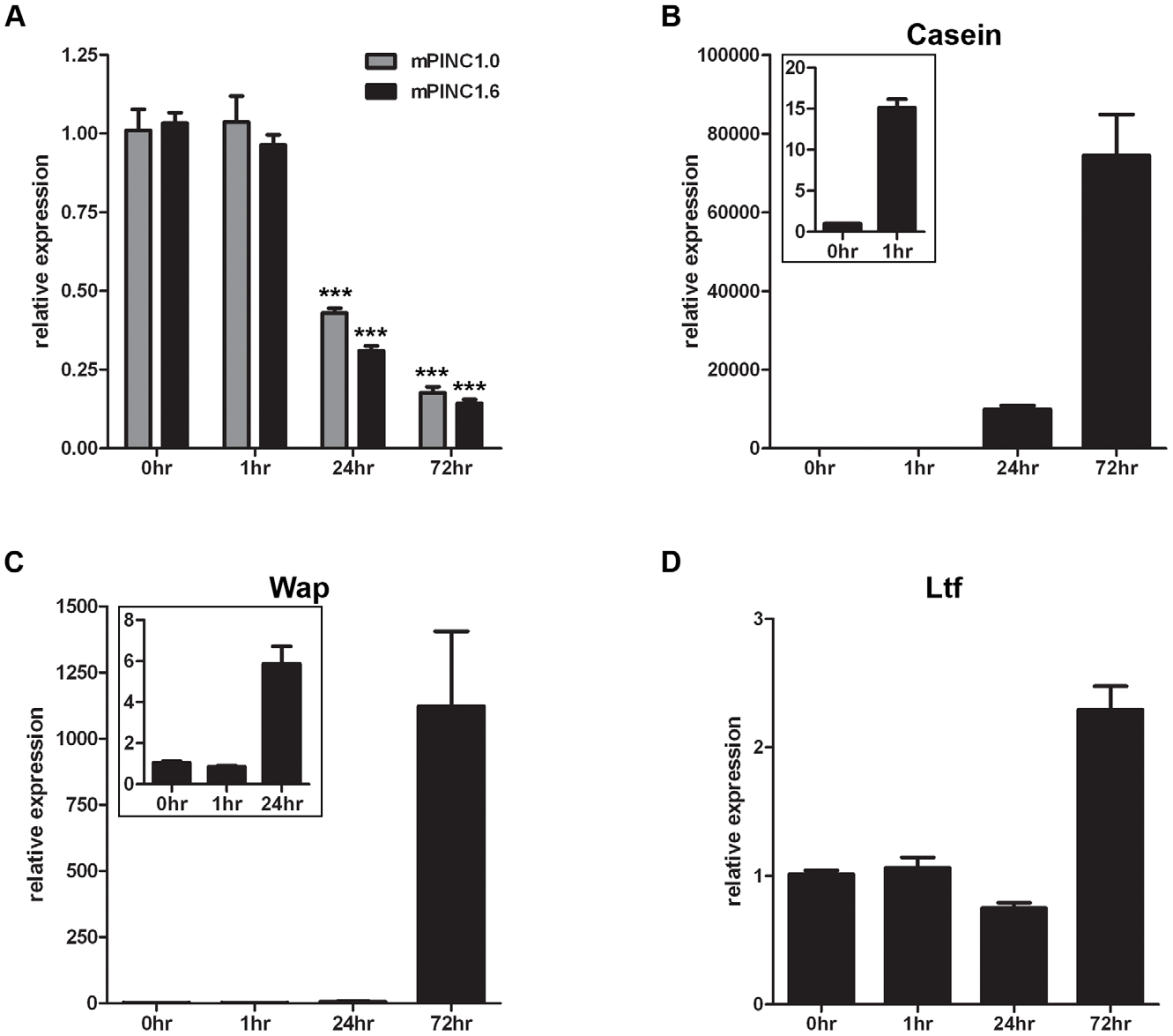
*mPINC* expression declines during lactogenic hormone induced differentiation of HC11 cells. (A–C) Confluent HC11 cells were treated with lactogenic hormones for 1, 24 and 72 hrs followed by RNA isolation to detect changes in gene expression. Target genes were normalized to *Gapdh* and set relative to levels in untreated HC11 cells. Data are presented as mean ± SEM (n = 3). (A) qPCR shows *mPINC1.0* and *mPINC1.6* expression decreases following 24 and 72 hrs of hormone treatment. (B) qPCR shows *Csn2* levels begin to rise after 1 hr of hormone treatment and continue to rise at 24 and 72 hrs. (C) qPCR shows *Wap* expression levels rise following 24 hrs of hormone treatment and continue to rise after 72 hrs. (D) *Ltf* expression levels rise following 72 hrs of lactogenic hormone treatment.

During the transition from late pregnancy to lactation, alveolar cells undergo the final step of alveologenesis called secretory activation, which is necessary to produce and secrete high levels of milk proteins. Since a significant drop in *mPINC* expression levels temporally coincides with this important process, we decided to test whether *mPINC* plays a functional role in the terminal differentiation of alveolar cells. To do this, we used an *in vitro* model of lactogenic differentiation in which HC11 cells, a mouse mammary epithelial cell line derived from the Comma-D cell line originally isolated from a mid-pregnant Balb/c mouse, were treated with the lactogenic hormones hydrocortisone, insulin and prolactin to induce differentiation [42], [43]. As a read-out for differentiation, we measured the expression levels of three milk protein genes, beta-casein (*Csn2*), whey acidic protein (*Wap*) and lactotransferrin (*Ltf*). After one hour of hormone treatment, there was an increase in the expression of *Csn2*, which continued to rise at 24 and 72 hours (Figure 4B). *Wap* expression was unchanged after one hour, but significantly increased after 24 hours of hormone treatment and continued to rise at 72 hours (Figure 4C). *Ltf* expression levels only began to rise following 72 hours of hormone treatment (Figure 4D). *mPINC* expression was unchanged after one hour, but both *mPINC1.0* and *mPINC1.6* were reduced by more than 50% following 24 hours of lactogenic hormone treatment and by more than 75% after 72 hours of treatment (Figure 4A). These data are consistent with the downregulation of *mPINC* seen in early lactation and suggest that *mPINC* may play a functional role in regulating differentiation of alveolar cells. These results also indicate that HC11 cells are a suitable model to further explore the role of *mPINC* in regulating hormonally-induced lactogenic differentiation.

### Overexpression of *mPINC* inhibits differentiation

**Figure 5:**
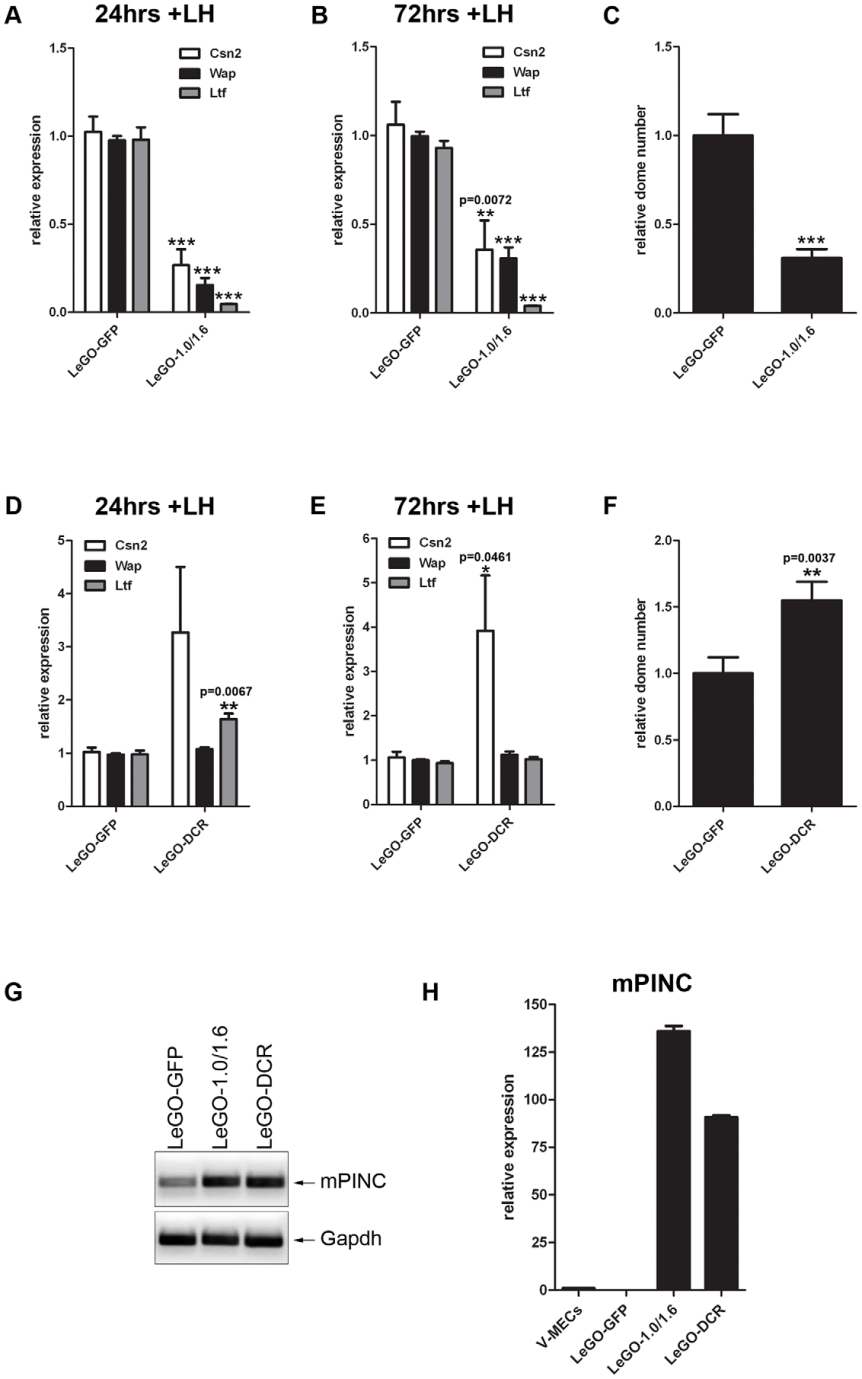
Overexpression of *mPINC* inhibits differentiation of HC11 cells. (A–C) After 24 (A) and 72 (B) hrs of hormone induction, LeGO-1.0/1.6 cells show reduced levels of *Csn2*, *Wap*, and *Ltf* expression by qPCR. Target genes were normalized to *Gapdh* and set relative to LeGO-GFP levels. Data are presented as mean ± SEM (n = 6). (C) Overexpression of *mPINC* also reduces formation of domes. Domes were counted at 48 hrs post-hormone treatment from nine 20× fields/experiment and data represent mean ± SEM set relative to the dome number in the LeGO-GFP control group (n = 6). (D–F) After 24 (D) and 72 (E) hrs of hormone induction, LeGO-DCR cells show increased levels of *Ltf* (at 24 hrs) and *Csn2*, but not *Wap*, expression compared to LeGO-GFP cells. Target genes were normalized to *Gapdh* and set relative to LeGO-GFP levels. Data are presented as mean ± SEM (n = 6). (F) Overexpression of the *DCR* mutant enhances dome formation compared to the control. Experiment was performed and analyzed as described in panel C. (G) RT-PCR shows *mPINC* overexpressed transcript in LeGO-1.0/1.6 and LeGO-DCR HC11 cells compared to endogenous levels in LeGO-GFP cells. (H) qPCR shows *mPINC* overexpressed levels are high and fairly equivalent relative to *mPINC* endogenous levels in MECs purified from virgin mammary gland (V-MECs). Target gene was normalized to *Gapdh*. Data are presented as mean ± SEM for three independent experiments.

The decline in *mPINC* levels observed during lactogenic differentiation *in vivo* and *in vitro* suggests that *mPINC* downregulation may be necessary to allow alveolar cells to undergo terminal differentiation. If this is true, then maintenance of high levels of *mPINC* should inhibit differentiation. To test this hypothesis, we generated HC11 cells overexpressing both *mPINC1.0* and *mPINC1.6* (LeGO-1.0/1.6), or a vector control (LeGO-GFP) using a lentiviral vector. We then treated the overexpressing cells with lactogenic hormones to induce differentiation. Following 24 and 72 hours of treatment, qPCR analysis showed that *mPINC* overexpressing cells expressed significantly lower levels of *Csn2*, *Wap* and *Ltf* mRNA compared to control cells (Figure 5A, 5B).

In addition to changes in gene expression, HC11 lactogenic differentiation is accompanied by morphological changes [43]. These changes include the formation of fluid-filled structures called domes that can be quantified as an indication of the extent of HC11 differentiation. Overexpression of *mPINC* caused a drastic reduction in the formation of dome structures compared to the control group (Figure 5C). Taken together, these results indicate that high levels of *mPINC* inhibit lactogenic differentiation of mammary epithelial cells, and that downregulation of *mPINC* may be required for terminal differentiation of alveolar cells in early lactation.

To determine whether the highly conserved regions of *mPINC* are necessary for its function as an inhibitor of HC11 differentiation, we generated a deletion mutant of *mPINC* (DCR) that lacked both terminal exons of *mPINC1.0* and *mPINC1.6*, including CR1 and CR2. We found that overexpression of the *mPINC* deletion mutant (LeGO-DCR) failed to inhibit lactogenic differentiation. Instead, the DCR overexpressing cells showed slightly increased levels of *Csn2* mRNA compared to control cells (LeGO-GFP) at both 24 and 72 hours after lactogenic hormone treatment, as well as increased *Ltf* expression at 24 hours, while *Wap* expression was unchanged (Figure 5D, 5E). Dome formation was also increased 1.5-fold (Figure 5F) compared to control cells. The increased markers of differentiation indicate that the DCR mutant could be interfering with endogenous *mPINC* function (see Discussion). However, more importantly, these results demonstrate that the conserved regions of *mPINC* are necessary for its function as an inhibitor of lactogenic differentiation.

To verify that the different phenotypes of the LeGO-1.0/1.6 and LeGO-DCR cells were not due to differences in overexpression levels, we performed RT-PCR and qPCR using RNA isolated from the two cell lines. RT-PCR analysis confirmed overexpression of *mPINC* compared to endogenous *mPINC* levels in control cells. In addition, qPCR analysis showed that the LeGO-1.0/1.6 and LeGO-DCR HC11 cells expressed *mPINC* at high (135-fold and 90-fold, respectively) and fairly equivalent levels compared to LeGO-GFP cells and MECs isolated from adult virgin mammary glands (Figure 5G, 5H). These data further suggest that the conserved regions of *mPINC* are necessary for its inhibitory effect on differentiation.

### Knockdown of *mPINC* increases differentiation of HC11 cells

**Figure 6:**
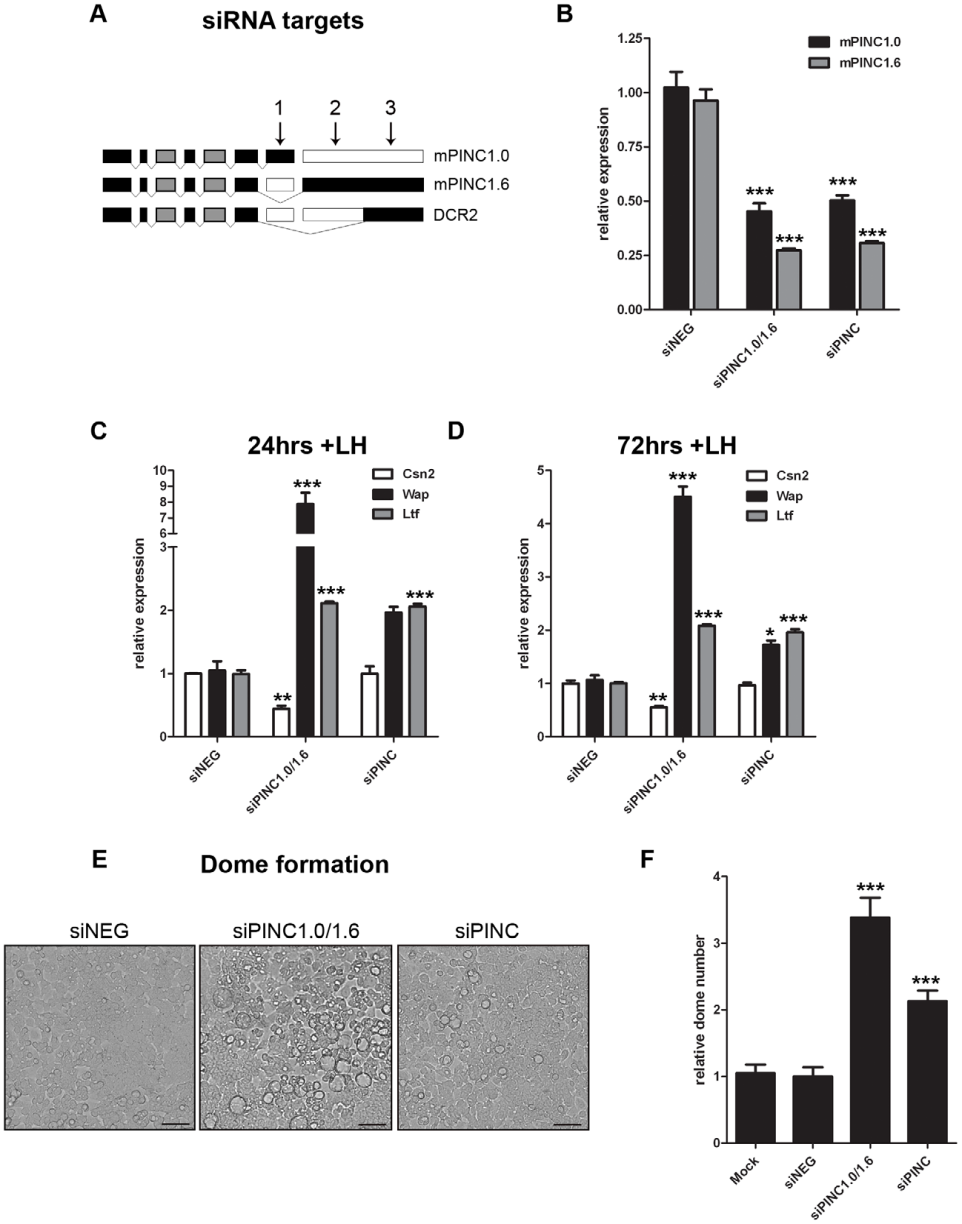
Knockdown of *mPINC* enhances differentiation of HC11 cells. (A, B) To target *mPINC1.0* and *mPINC1.6* (siPINC1.0/1.6), but not *DCR2*, siRNAs #1 and #2 were used in combination. To target all splice variants (siPINC), siRNAs #1 and #3 were used in combination. (A) Schematic showing siRNA targets of *mPINC* splice forms. (B) qPCR shows knockdown of *mPINC* 5 days post-transfection of siRNAs. Target genes were normalized to *Gapdh* and set relative to levels in the siNEG control transfected HC11 cells. (C, D) Knockdown of *mPNC* (siPINC1.0/1.6 and siPINC) splice forms increases *Wap* and *Ltf*, but not *Csn2*, expression at 24 (C) and 72 (D) hrs post-hormone induction. Target genes were normalized to *Gapdh* and set relative to levels in siNEG treated control cells. Data are presented as mean ± SEM (n = 3). (E, F) Knockdown of *mPINC* also increases dome formation compared to a control. (E) Representative brightfield images show domes following 48 hrs of hormone treatment. Scale bars represent 50 µm. (F) Domes were counted at 48 hours post-hormone treatment from nine 20× fields/experiment and data represent mean ± SEM set relative to the dome number in the siNEG treated control group (n = 6).

As *mPINC* overexpression inhibited differentiation of HC11 cells, we sought to determine if a reduction in *mPINC* levels could enhance differentiation. Because there is an endogenous *mPINC* isoform that lacks the conserved regions, *DCR2*, it was of interest to ascertain if its function is distinct from the other *mPINC* splice forms. Therefore, we used pairs of siRNAs to target *mPINC1.0* and *mPINC1.6* only (siPINC1.0/1.6) or all splice forms of *mPINC*, including the *DCR2* isoforms (siPINC) (Figure 6A, 6B). After transfection with siRNAs, the cells were treated with lactogenic hormones to induce differentiation. At 24 and 72 hours post-treatment, knockdown of *mPINC1.0* and *mPINC1.6* resulted in increased expression of *Wap* and *Ltf* mRNA (Figure 6C, 6D). Knockdown of all splice forms of *mPINC* resulted in increased *Ltf* levels, but only a slight increase of *Wap* expression at 72 hours post-hormone treatment. Unexpectedly, knockdown of *mPINC1.0* and *mPINC1.6* reduced *Csn2* levels, while knockdown of all *mPINC* isoforms had no effect on *Csn2*. Due to the discrepancies in these differentiation markers, we looked at the effect of knockdown on the morphological assay of differentiation. Knockdown of *mPINC1.0* and *mPINC1.6* increased dome formation 3.5-fold in treated cells, while knockdown of all *mPINC* splice forms increased dome formation 2-fold (Figure 6E, 6F). Taken together, these data provide support for the role of *mPINC* in inhibiting lactogenic differentiation. Interestingly, knockdown of all isoforms of *mPINC*, including *DCR2*, slightly diminished the effect on differentiation. This suggests that the endogenously expressed *DCR2*, that lacks the most conserved regions, may act to reduce the inhibitory effects of *mPINC1.0* and *mPINC1.6* on alveolar differentiation.

### *mPINC* interacts with PRC2 *in vitro*

**Figure 7:**
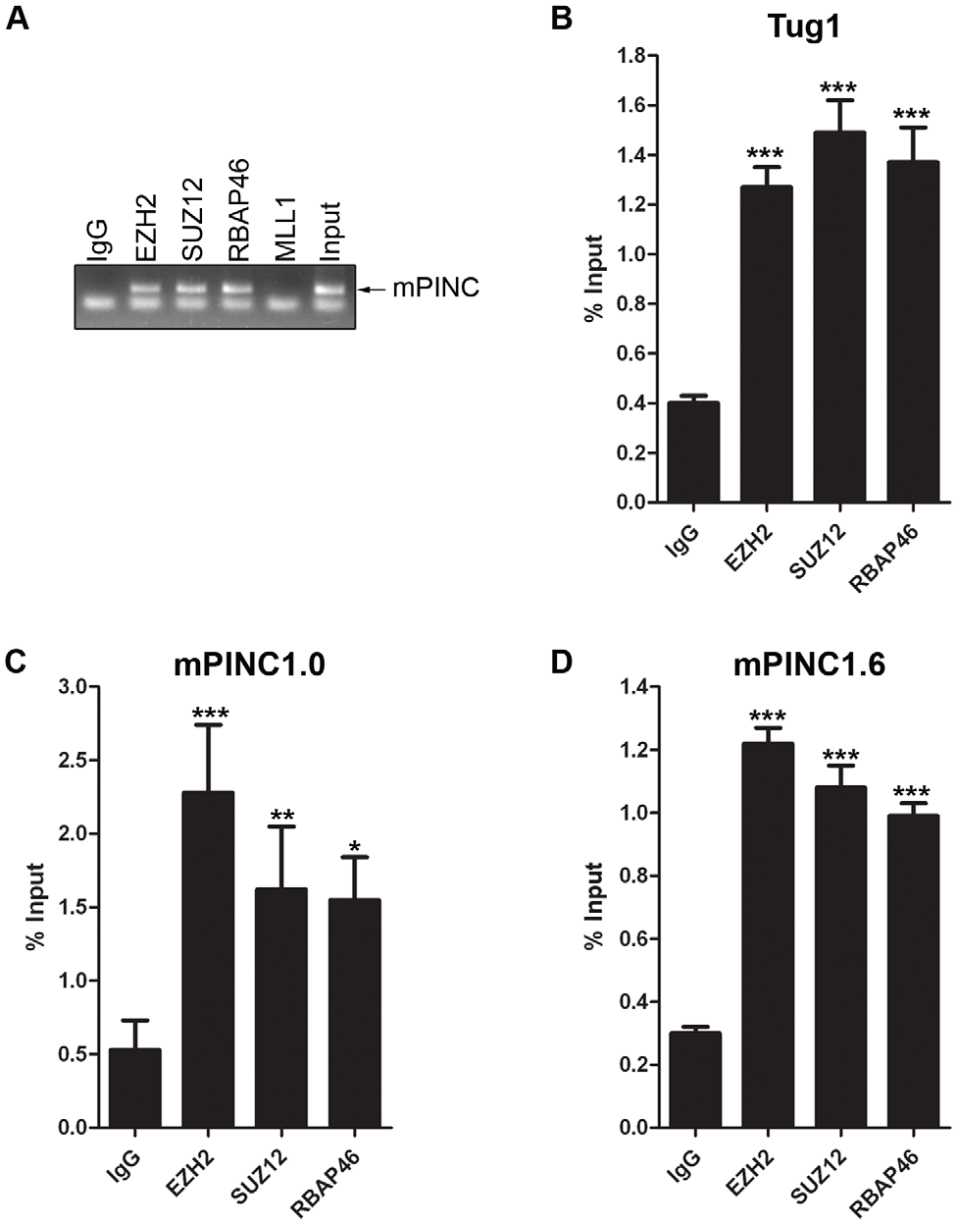
*mPINC* interacts with PRC2 in HC11 cells. (A–D) RIP assays were performed with HC11 cells using antibodies to PRC2 members including, EZH2, SUZ12 and RbAp46. An MLL1 antibody was used as a negative control and an lncRNA known to interact with PRC2, *Tug1*, was used as a positive control. RNA was isolated from pull downs to detect associated RNAs. (A) RT-PCR shows *mPINC* is associated with PRC2 members, but not MLL1. (B–D) qPCR shows the amount of *Tug1* (B), *mPINC1.0* (C) and *mPINC1.6* (D) transcript associated with each protein as a percentage of input RNA levels. Data represent mean ± SD (n = 3).

The previous experiments strongly suggest that *mPINC* plays a role in regulating alveolar differentiation. However, the mechanisms responsible are unknown. Many lncRNAs interact with chromatin-remodeling complexes such as PRC2. Interestingly, a member of PRC2, *RbAp46*, was identified in the same screen as *mPINC*, and both genes are expressed in the alveolar cells during pregnancy and in the regressed lobules of the post-involuted gland. Thus, to investigate a potential interaction with *mPINC* in HC11 cells, we performed RNA immunoprecipitation (RIP) assays using antibodies to members of PRC2, including enhancer of zeste homolog 2 (EZH2), suppressor of zeste 12 homolog (SUZ12) and RbAp46. RT-PCR using primers to detect *mPINC* showed that there was a specific interaction with all three PRC2 complex members, but not with mixed-lineage leukemia (MLL1), a member of the activating trithorax complex (Figure 7A). By qPCR, we found that both *mPINC1.0* and *mPINC1.6* splice forms interacted with PRC2 members (Figure 7C, 7D). *Tug1*, an lncRNA previously shown to interact with PRC2, was used as a positive control (Figure 7B). Together, these data indicate that *mPINC* interacts with PRC2 in HC11 cells, providing a potential mechanism of the inhibitory effect of *mPINC* on lactogenic differentiation.

### Overexpressed *mPINC* and the DCR mutant interact with PRC2

**Figure 8:**
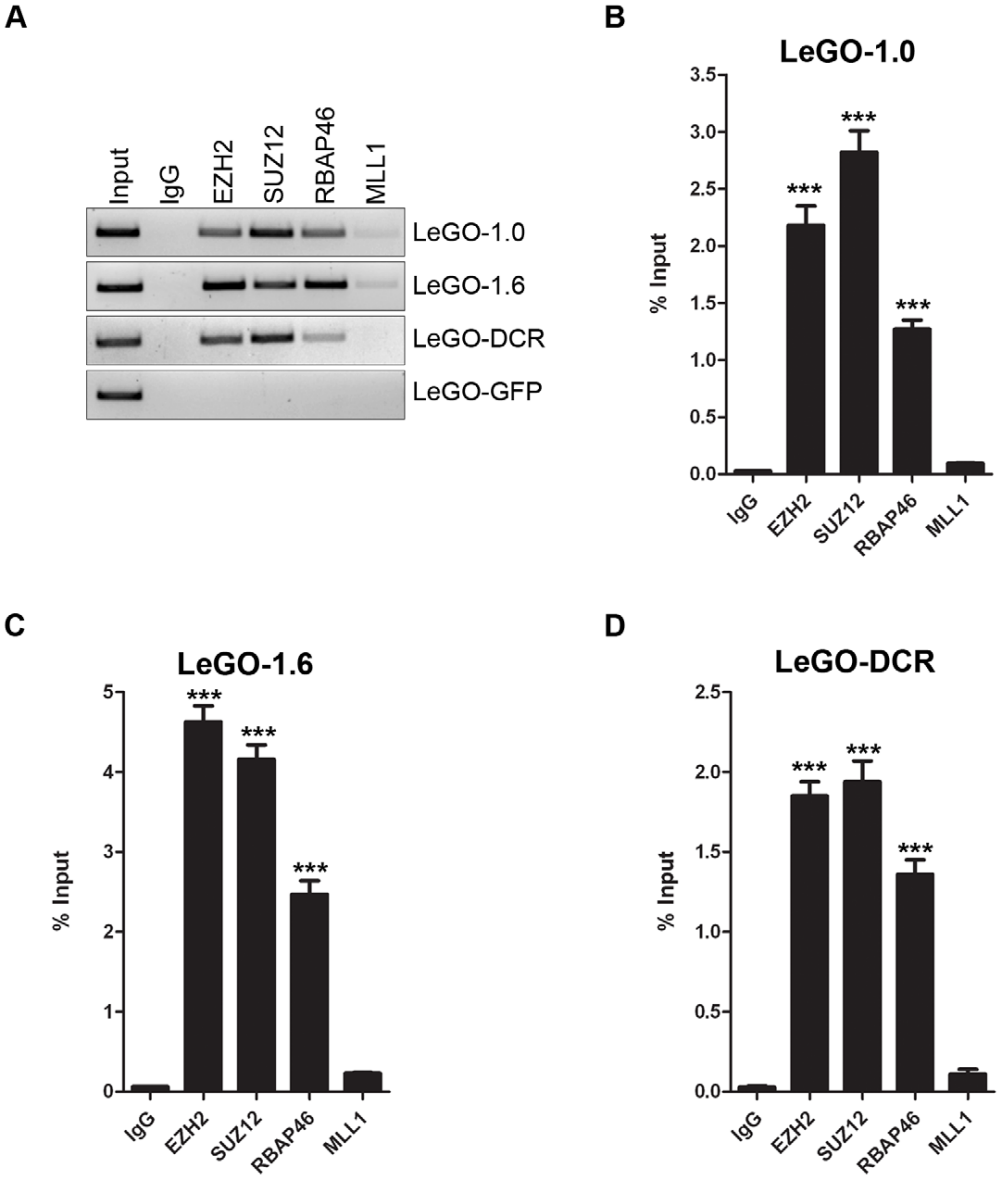
Overexpressed *mPINC* transcripts interact with PRC2 in HC11 cells. (A–D) RIP assays were performed with *mPINC* overexpressing HC11 cell using antibodies to PRC2 members including, EZH2, SUZ12 and RbAp46. An MLL1 antibody was used as a negative control. (A) RT-PCR shows that overexpressed *mPINC1.0*, *mPINC1.6* and the *DCR* mutant interact with PRC2 members, but not IgG or MLL1. (B–D) qPCR shows the amount of *mPINC1.0* (B), *mPINC1.6* (C) and DCR (D) transcript associated with each protein as a percentage of input RNA levels. Data represent mean ± SD (n = 3).

To show that the overexpressed *mPINC* transcripts are functional, we sought to determine if they, like endogenous *mPINC*, interact with PRC2. Using a reverse primer to recognize a portion of the lentiviral vector that remains attached to the *mPINC* transcripts, we found that overexpressed *mPINC1.0* and *mPINC1.6* interacted with PRC2, suggesting that they are able to function like endogenous *mPINC* (Figure 8A). RT-PCR also showed that the DCR mutant retained the ability to interact with PRC2, providing evidence that the 5′ end of the RNA likely interacts with PRC2 and that the highly conserved regions at the 3′ end are not necessary for this interaction. qPCR analysis showed that the interaction of the overexpressed transcripts is specific to PRC2 (Figure 8B–8D). These data also suggest that the inability of the overexpressed DCR form of *mPINC* to inhibit lactogenic differentiation is not due to loss of binding to PRC2, but perhaps to the inability of the DCR mutant to interact with an additional unknown interacting protein or nucleic acid sequence target of *mPINC*.

### *mPINC* interacts with PRC2 *in vivo*

**Figure 9:**
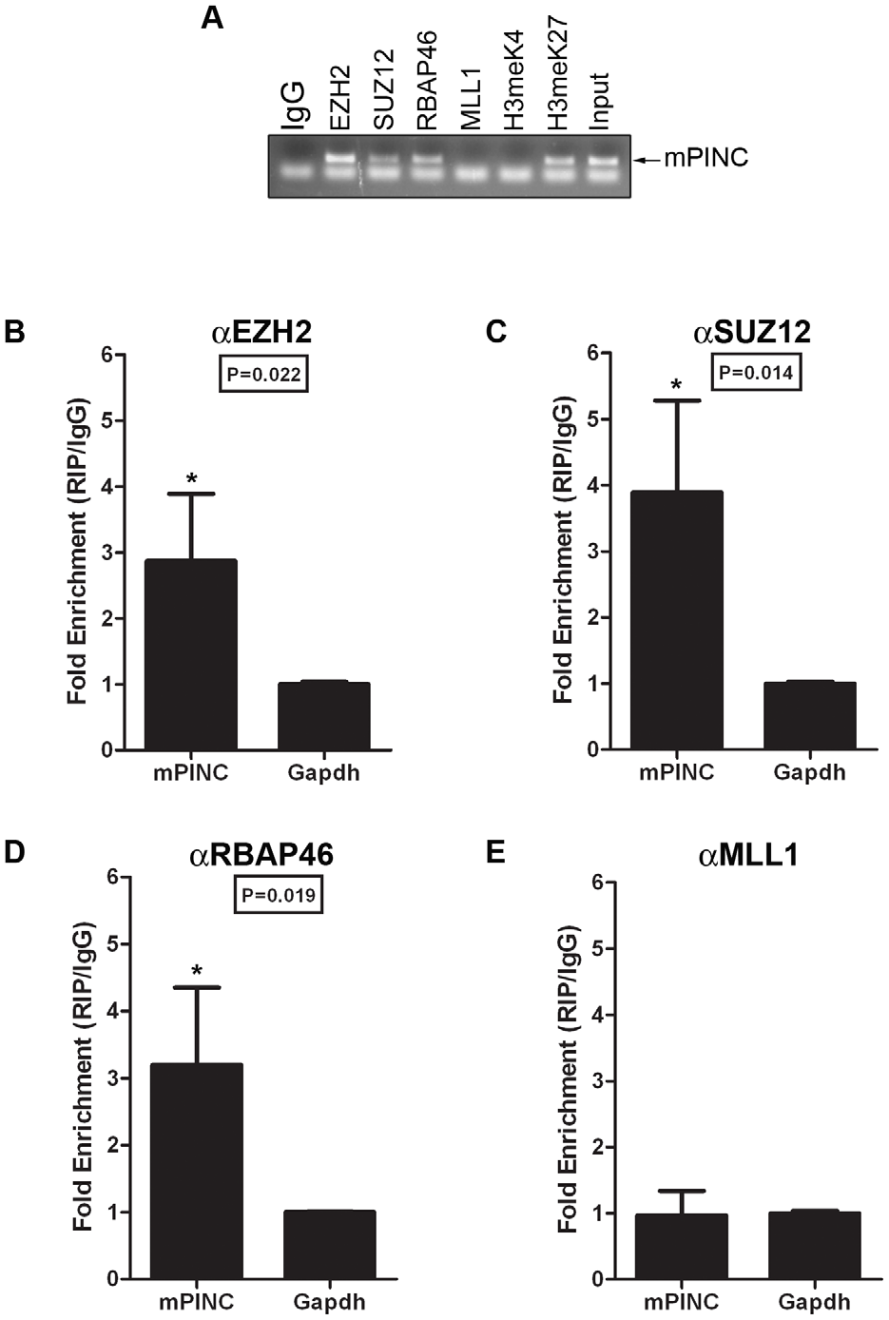
*mPINC* associates with PRC2 in the 16-day pregnant mammary gland. (A–D) RIP assays were performed with MECs purified from mammary glands at day 16 of pregnancy using antibodies to PRC2 members and its associated histone modification, H3meK27. Antibodies to MLL1 and its associated histone modification, H3meK4, were also used as negative controls. (A) RT-PCR shows *mPINC* is associated with PRC2 members and H3meK27. *mPINC* does not associate with MLL1 or H3meK4. (B–D) qPCR shows fold enrichment of *mPINC* transcript levels associated with EZH2 (B), SUZ12 (C) RpAp46 (D) and MLL1 (E) relative to *Gapdh* levels. Data represent mean ± SD (n = 3).

To determine whether *mPINC* also interacts with PRC2 *in vivo*, MECs were purified from mice at day 16 of pregnancy. RIP assays were performed using antibodies to PRC2 members and to the associated histone modification, H3meK27. As negative controls, antibodies to MLL1 and to the associated activating histone modification, H3meK4 were used. RT-PCR showed that *mPINC* is associated with PRC2 members and H3meK27, but *mPINC* did not interact with MLL1 or H3meK4 in MECs isolated from pregnant mice (Figure 9A). Further qPCR analysis showed that the interaction between *mPINC* and PRC2 members was enriched relative to the housekeeping gene, *Gapdh*, and that this enrichment was specific to PRC2 members and not to MLL1 (Figure 9B–9E). These results suggest that the interaction between *mPINC* and PRC2 is relevant *in vivo*, as well as *in vitro*, where it potentially plays a role in late pregnancy and involution to maintain alveolar progenitors by inhibiting terminal differentiation.

### Microarray analysis reveals potential targets of *mPINC*-PRC2

**Figure 10:**
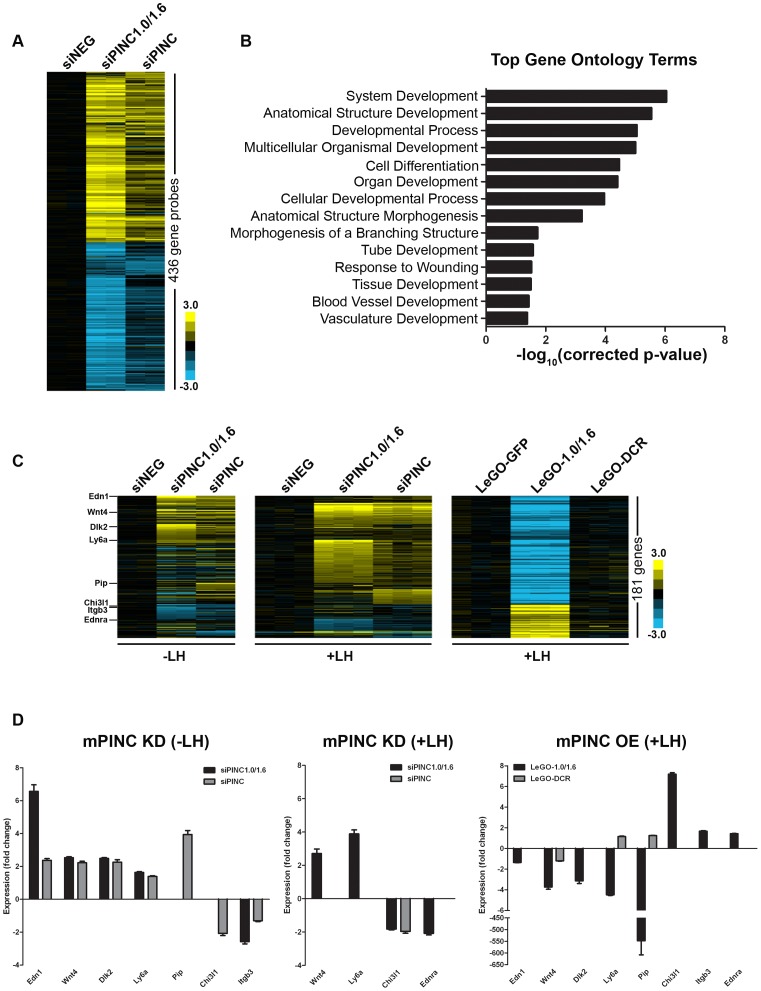
Microarray identifies potential targets of *mPINC* in HC11 cells. (A) Heat map showing transcript profiling of biological replicates of control siNEG cells, siPINC1.0/1.6, and siPINC 5 days post-siRNA transfection ( p<0.01 and fold change >1.8 in either siPINC1.0/1.6 or siPINC relative to siNEG). (B) Graph indicating the most significantly enriched gene ontology terms in the *mPINC* knockdown data set. (C) Three heat map panels depicting genes that are differentially expressed between either undifferentiated (−LH) *mPINC* knockdown cells (left panel) or differentiated (+LH) *mPINC* knockdown cells (middle panel) and differentiated (+LH) *mPINC* overexpression cells (right panel). 181 genes were found to be differentially regulated by *mPINC* (141 genes are upregulated by knockdown and downregulated by overexpression, while 40 genes are downregulated by knockdown and upregulated by overexpression at a p<0.01 and fold change >1.4, both for the overexpression and either knockdown group in the opposite direction). Genes whose expression was validated by qPCR are indicated in the heat map on the left. (D) qPCR verified differential expression of genes in *mPINC* knockdown (KD) compared to overexpression (OE) cells. qPCR was performed on biological triplicates, normalized using *Gapdh*, and shown as fold change compared to the negative control (either siNEG or LeGO-GFP).

To identify potential targets of *mPINC*, we performed oligonucleotide microarray analysis on undifferentiated HC11 cells following *mPINC* knockdown (siPINC1.0/1.6 and siPINC). *mPINC* depletion resulted in the disrupted expression of 303 unique annotated genes (436 gene probes, shown in Table S1) (Figure 10A). Interestingly, loss of *mPINC1.0* and *mPINC1.6* (siPINC1.0/1.6) resulted in more gene expression changes than loss of all *mPINC* splice forms (siPINC). In addition, the array data identified unique genes whose expression was changed by either siPINC1.0/1.6 knockdown or siPINC knockdown alone, further indicating distinct functions for the *mPINC* splice forms. However, many of the genes altered by siPINC1.0/1.6 knockdown were also altered by siPINC knockdown, yet to a lesser extent. These results are in agreement with, and may help explain, the reduced effect of siPINC knockdown on lactogenic differentiation compared to siPINC1.0/1.6 knockdown. Gene ontology (GO) analysis of altered genes in the *mPINC* knockdown cells demonstrated GO terms were significantly enriched for developmental processes and differentiation (Figure 10B) (Table S2). GO terms more specifically related to mammary development, such as tube development and branching morphogenesis, were also enriched. These data indicate that *mPINC* generally regulates genes involved in development and differentiation.

To better determine targets of *mPINC* that may be involved in regulating alveolar differentiation, we also performed microarray analysis on *mPINC* knockdown and *mPINC* overexpression cells treated with lactogenic hormones. As knockdown and overexpression of *mPINC* resulted in opposing phenotypes on differentiation, we looked for genes that were altered in opposite directions by knockdown and overexpression. For the knockdown analysis, we used the array data from undifferentiated (−LH) and differentiated (+LH) HC11 cells to reveal hormonally-regulated targets of *mPINC*. This analysis narrowed the list of potential *mPINC* targets to 181 genes (Figure 10C) (Table S3). Almost 80% of the identified genes (141 genes) were upregulated in the *mPINC* knockdown cells and downregulated in the overexpression cells, providing evidence that *mPINC* functions as part of a repressive PRC2 complex. This analysis also revealed several differentially expressed genes with known or suggested roles in mammary gland development and alveolar differentiation, including members of the Wnt and Notch signaling pathways. Genes that were upregulated by mPINC knockdown and downregulated by overexpression include endothelin 1 (*Edn1*), *Wnt4*, delta-like 2 (*Dlk2*), *Ly6a* (or *Sca1*) and prolactin-induced protein (*Pip*). Conversely, genes that were downregulated by mPINC knockdown and upregulated by overexpression include chitinase 3-like 1 (*Chi3l1*), integrin-beta 3 (*Itgb3* or *CD61*), endothelin receptor a (*Endra*) and of course, *mPINC* itself. The differential expression of each of these genes was confirmed by qPCR (Figure 10D). The genes identified by microarray provide mechanistic insight into how *mPINC*-PRC2 may regulate alveolar differentiation as a repressive complex in the late pregnant and early involuting mammary gland.

### *mPINC* splice forms have common, stable hairpin structures that may interact with PRC2

**Figure 11:**
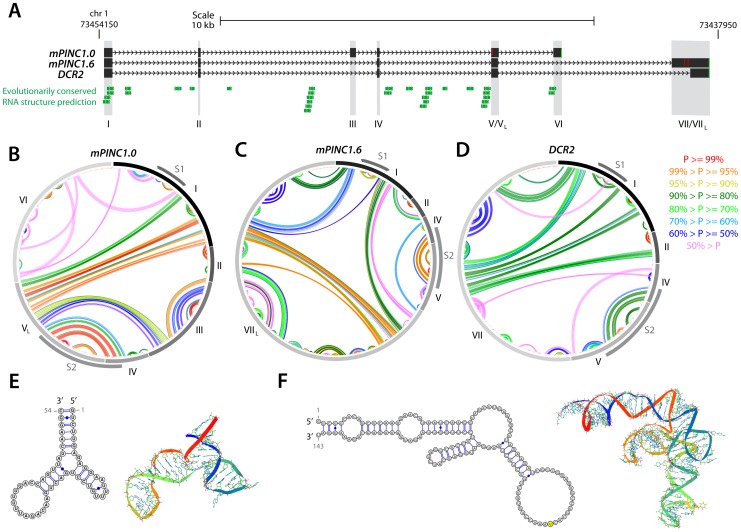
Structural analysis of the *PINC* locus. (A) Genomic representation of the *PINC* locus showing alternate isoforms *mPINC1.0*, *mPINC1.6* and *DCR2*. Regions of evolutionarily conserved RNA structures were predicted by SISSIz (see Methods). (B–D) Circle plot secondary structure representations of interacting nucleotides in *mPINC1.0* (B), *mPINC1.6* (C) and *DCR2* (D). The probability of the interactions is color-coded according to the legend (right). Secondary structures were computed using the RNAstructure software (see Methods). (E–F) High-confidence localized hairpin structures that represent possible protein-interaction sites and their most stable 3D representations, as modelled by MC-FOLD/MC-SYM. The corresponding regions in B–D are indicated by S1, for structure 1 (E) and by S2, for structure 2 (F) grey arcs.

Although there is increasing evidence that many lncRNAs interact with PRC2 to direct their activity towards particular regions in the genome, the mechanism of this interaction remains a mystery. Recent studies identified double-stem loop structures in the *RepA* and *Hes1-as* lncRNAs that are thought to interact directly with the EZH2 subunit of PRC2 [20], but whether this will be a motif common to all PRC2-interacting lncRNAs is not yet known. To determine whether *mPINC* contained any structures that may interact with PRC2, we used the RNAstructure software to predict the secondary structure interactions of the *mPINC* isoforms *mPINC1.0*, *mPINC1.6* and *DRC2*. Using SISSIz [44], we also looked for regions within the *mPINC* locus that showed significant evolutionary conservation of RNA secondary structure in all vertebrates (Figure 11A). As a result, we found two double hairpin regions that occurred in all three isoforms, indicated in the circular plots as structure 1 (S1) and structure 2 (S2) (Figure 11B–11D). The first is a 53 nt Y-shaped structure arising from exon 1, which showed a high level of evolutionary conservation (Figure 11E). This structure appears similar to that identified in *RepA* and we therefore propose that it may be a good candidate site for PRC2-interaction. The second structure is a 143 nt double hairpin that arises from exons 4 and 5 (Figure 11F). Although this region did not contain signatures of evolutionary conservation, the structure was predicted to fold with high probability. Given the high stability of the second site, we anticipate that it will also play an important role in the function of *mPINC*, possibly as an additional protein-interaction site. These data show that the 5′ region of *mPINC*, which is sufficient for interacting with PRC2, contains evolutionarily conserved stable hairpin structures that are reminiscent of double stem-loop structures of lncRNAs that have previously been shown to bind the EZH2 subunit of PRC2.

## Discussion

Previously, we identified *PINC* and *RbAp46*, a member of the chromatin-modifying PRC2 complex, as genes that are elevated in alveolar cells of the pregnant and involuting rat mammary gland. *In vitro* studies of *mPINC* showed potential roles in regulating survival and cell-cycle progression of mammary epithelial cells, and it was further concluded that *mPINC* was a marker of alveolar cell fate. In this study, we extend our previous observations and demonstrate a functional role for *mPINC* in regulating alveolar development. First, we showed that *mPINC* is highly expressed in alveolar cells in the mouse mammary gland during pregnancy and downregulated in early lactation. Similarly, we found that *mPINC* levels decline upon lactogenic hormone-induced differentiation of HC11 cells. To investigate the functional significance of *mPINC* downregulation, we performed gain- and loss-of-function studies using HC11 differentiation assays. Overexpression of *mPINC* blocked lactogenic differentiation, while knockdown of *mPINC* resulted in enhanced markers of differentiation. As previous data showed that *PINC* and *RbAp46* are co-regulated and co-localized in the rat mammary gland, we sought to determine a potential interaction in mammary epithelial cells. We provide evidence that *mPINC* is associated with RbAp46 and other PRC2 members in mammary epithelial cells both *in vitro* and *in vivo*. Finally, microarray analysis of *mPINC* knockdown and overexpression cells provided substantial insight into the putative gene networks modified by *mPINC*-PRC2. These results suggest that *mPINC* likely functions epigenetically as part of a chromatin-modifying PRC2 complex in the post-pubertal mammary gland to negatively regulate mammary alveolar differentiation.

As many lncRNAs show tissue-specific expression patterns and spatiotemporal regulation during development, it is not surprising that several lncRNAs regulate key developmental processes such as cell-fate commitment and differentiation. Large-scale analysis has revealed differentially expressed lncRNAs during mouse embryonic stem (ES) cell differentiation, thus identifying novel lncRNAs associated with either pluripotency or various stages of differentiation [45]. In addition, several lncRNAs regulate specific processes of development, such as germ layer specification [46], muscle cell differentiation [47], and terminal differentiation of erythroid cells [48] and skin cells [49]. Recently, a developmentally regulated lncRNA, *Zfas1*, was identified in the post-pubertal mouse mammary gland [50]. *Zfas1* exhibits a 10-fold decrease between pregnancy and lactation and is downregulated in lactogenic hormone-induced differentiation of HC11 cells. Like *mPINC*, knockdown of *Zfas1* results in enhanced lactogenic differentiation of HC11 cells. However, the molecular mechanisms that regulate the function of *Zfas1* are unknown. In this study, we found that *mPINC* regulates mammary alveolar differentiation and we demonstrate, for the first time, the interaction of an lncRNA with PRC2 in mammary epithelial cells.

PRC2 has a well-known role in maintaining cellular identity by stably repressing different sets of genes during differentiation and development [51], [52]. Historically, PRC2 has been regarded as a critical regulator of gene expression during embryonic development [53]. However, mounting evidence suggest that PRC2 is also involved in maintaining homeostasis in adult tissues by regulating the balance between self-renewal and differentiation of specific progenitor populations [54]–[56]. For example, loss of the enzymatic subunit of PRC2, EZH2, in either cortical or epidermal progenitor cells results in accelerated differentiation, while overexpression of EZH2 in myoblasts inhibits muscle cell differentiation. Intriguingly, our data suggest an additional role for PRC2, in association with the lncRNA *mPINC*, in maintaining alveolar progenitors by repressing genes necessary for alveolar differentiation.

Precisely how PRC2 targets specific loci to control the fate of a particular cell is unknown. However, increasing evidence shows that lncRNAs may be, in part, responsible. The earliest and best-characterized example is the silencing of the X chromosome during dosage compensation by PRC2 recruitment, which was recently shown to be mediated in *cis* by a 1.6 kilobase lncRNA called *RepA* [22]. More recently, several more lncRNAs have been shown to be required for the targeting of PRC2 to specific loci in *cis*, such as *Kcnq1ot1* silencing of the *Kcnq1* imprinted locus and *Anril* repression of the *p15^INK4B^* locus [23], [24]. In this study, we showed that *mPINC* interacts with PRC2 *in vitro* and *in vivo* and microarray analysis of mammary epithelial cells following *mPINC* modulation revealed 181 potential target genes. Analysis of these target genes revealed only one gene proximal to the *mPINC* locus on chromosome 1, *Igfbp2*. *Igfbp2* is located 400 kilobases downstream of *mPINC* and is differentially regulated during mammary development [57]. IGFBP2 levels decline throughout pregnancy and lactation with the most significant reduction occurring at day 2 of involution. Interestingly, *mPINC* levels are highest at day 2 of involution and may play a role in the repression of *Igfbp2* at this time point. However, the function of *Igfbp2* during involution, as well as during other stages of mammary development, is unknown.

LncRNAs also recruit PRC2 in *trans* as evidenced by the lncRNA *HOTAIR*, which is transcribed from the *HOXC* locus but is required to target PRC2 to the *HOXD* locus on a different chromosome [13]. *mPINC* may operate like *HOTAIR* and target genes in *trans* to regulate alveolar differentiation. Analysis of potential *mPINC* target genes showed that they are distributed throughout the genome, with the exception of *Igfbp2*, indicating that *mPINC* is most likely regulating genes in *trans*. The most upregulated gene in *mPINC* knockdown cells, *Edn1*, is an important cell fate determinant during embryogenesis [58], [59]. It is highly expressed in the mammary gland during lactation and is also induced in HC11 cells treated with lactogenic hormones [60]. The receptor for *Edn1*, *Ednra*, was downregulated by *mPINC* modulation, thus emphasizing the potential importance of this pathway. Another gene upregulated upon *mPINC* loss was *Wnt4*. *Wnt4* is induced by progesterone during pregnancy and is required for proper lobuloalveolar development [61]. *Wnt4* is also required for hormone-induced differentiation of HC11 cells [62]. The Notch signaling inhibitor *Dlk2* [63] was also induced in the *mPINC* knockdown cells, while a positive regulator of Notch signaling, *Mindbomb2* (*Mib2*) [64], was oppositely regulated. Active Notch signaling in mammary epithelial cells promotes luminal cell fate, while constitutive Notch signaling in the luminal cells during pregnancy inhibits alveolar differentiation, suggesting that repression of Notch activity is required for alveolar differentiation [65]. *Chi3l1* (or *Ykl40*) was downregulated by *mPINC* knockdown and upregulated by overexpression. Treatment of mammary epithelial cells with recombinant YKL40, or ectopic expression of *Ykl40* in mammary epithelial cells, inhibits lactogenic hormone-induced differentiation [66]. *Chi3l1* (or *Ykl40*) is one of the most highly induced genes during involution and could function downstream of *mPINC* to maintain alveolar progenitors in the involuted gland. The identification of these potential *mPINC*-PRC2 target genes provides plausible mechanisms for the impact of *mPINC* on alveolar differentiation.

The microarray data also provided additional evidence for the differentiation status of the HC11 cells following *mPINC* knockdown and overexpression. There was some variability in the effect of *mPINC* knockdown on markers of lactogenic differentiation, as *Csn2* levels were not increased similar to *Ltf* and *Wap* levels. The expression profiling, however, identified additional milk protein genes, as well as other lactation-induced genes, that were differentially expressed following *mPINC* knockdown and overexpression. These genes, which include *Cp*, *Mal*, *Clu*, *Csn3*, *Pip* and *Cidea*, were all induced by *mPINC* knockdown and decreased by overexpression [67], [68]. Additionally, overexpression of the *mPINC* DCR mutant not only failed to inhibit differentiation, but also showed enhanced expression of some markers of differentiation, suggesting the possibility that it acts in a dominant-negative manner. However, expression profiles of LeGO-DCR overexpressing cells clearly showed that they were very similar to the LeGO-GFP control cells, suggesting that the DCR mutant is not interfering with endogenous *mPINC* function. Thus, the microarray data provides further support for the hypothesis that *mPINC* is necessary for maintaining the undifferentiated state of mammary epithelial cells, and that the conserved regions are necessary for this function.

Lastly, the robust enrichment of *mPINC* in the luminal and alveolar progenitor populations further substantiates its role in maintaining cells in an undifferentiated state. Previous characterization of luminal subpopulations using CD61 and Sca-1 have shown that the luminal progenitors are CD61^+^ and Sca-1^−^, while the more differentiated, mature luminal cells are CD61^−^ and Sca-1^+^ [38]. Gene expression profiling showed that depletion of *mPINC* results in a reduction in *Itgb3* (or *CD61*) levels and an increase in *Ly6a* (or *Sca1*). In addition, *mPINC* knockdown also resulted in higher *Wnt4* levels, another marker of mature luminal cells. These data suggest that by decreasing levels of *mPINC*, luminal progenitor cells are pushed toward a more differentiated state. Interestingly, a recent study identified a novel lncRNA called *ANCR* (anti-differentiation ncRNA) that is enriched in epidermal progenitors [49]. Like *mPINC*, loss of *ANCR* leads to induction of differentiation genes, suggesting *ANCR* is required to maintain epidermal progenitors in an undifferentiated state. Taken together, these studies provide additional evidence for the important role of lncRNAs in the maintenance of cellular homeostasis across multiple tissues.

Our current work used a well-characterized *in vitro* model for functional characterization of *mPINC* in alveologenesis. Future studies should employ loss- and gain-of-function mouse models to further investigate the role of *mPINC in vivo* during pregnancy and involution. We also provided evidence for an interaction between *mPINC* and PRC2 and identified putative stable secondary structures that may be required for this interaction. Mutations to disrupt these stable hairpin structures should be generated to determine the structure-function relationships necessary for PRC2 binding. Finally, we identified potential targets of *mPINC*-PRC2, several of which have known or suggested roles in regulating alveologenesis. It will be critical to verify that these loci are actually targeted by *mPINC*-PRC2 to provide insight into how this repressive complex regulates the stepwise differentiation of alveolar cells.

We propose a model where *mPINC* and RbAp46 are elevated during pregnancy and involution and interact to repress genes necessary for alveolar differentiation, thereby preserving a population of alveolar progenitors for subsequent pregnancies. *PINC* and *RbAp46* were originally found in a screen to identify genes that are persistently altered in the parous mammary gland in an effort to determine the molecular mechanisms that govern the protective effect of an early pregnancy against tumorigenesis [35]. Future studies to better understand the epigenetic regulation of differentiation and development in the post-pubertal mammary gland will likely elucidate potential mechanisms that contribute to reduced tumorigenesis in the parous breast.

## Materials and Methods

### Animal care

This study was performed in strict accordance with the recommendations in the Guide for the Care and Use of Laboratory Animals of the National Institutes of Health. The animal research protocol was approved by the Institutional Animal Care and Use Committee of Baylor College of Medicine (AN-504). All mice used in this study were maintained and euthanized under the strict guidelines of the Institutional Animal Care and Use Committee of Baylor College of Medicine. Balb/c mice were obtained from Harlan Laboratories. For mammary gland developmental time points, mating pairs were co-housed in the afternoon and females were checked for a vaginal plug the following morning. For involution time points, pups were force-weaned after 10 days of lactation.

### Preparation of single mammary cell populations

The third, fourth, and fifth mammary fat pads were harvested from 8–10 week old virgin or 16 day pregnant Balb/c mice. The glands were subsequently weighed and minced into 1 mm×1 mm fragments using a McIlwain Tissue Chopper 800 series. Mammary organoids were derived by digesting the glands in DMEM/F12 containing 2 mg/ml Collagenase A (Roche) for 1 hr at 37°C with constant shaking at 150 rpm. Organoids were isolated from the digested preparation by differential centrifugation, consisting of a sequence of 4 to 5 brief centrifugation steps (5 sec pulses) at 1500 rpm to enrich for mammary organoids and remove contaminating stromal cells. The organoids were then resuspended in 0.25% Trypsin-EDTA for 5 min, washed, and filtered through a 0.40 µm cell strainer to obtain a single mammary epithelial cell preparation. Single cells were washed 2–3 times with HBSS prior to cell surface staining for flow cytometry or fixation for RNA immunoprecipitation.

### Cell sorting

Mammary epithelial cells were resuspended at a concentration of 1×10^8^ cells/ml in HBSS supplemented with 10 mM HEPES and 2% FBS. This cell suspension was depleted of lineage positive cells (CD45, Ter119, CD31, and BP-1) using the EasySep Mouse Epithelial Cell Enrichment Kit (Stem Cell Technologies). Mammary epithelial cells were subsequently resuspended at a density of 1×10^7^ cells/ml and stained with anti-mouse CD24 PE (Stem Cell Technologies, 1∶100), anti-CD49f FITC (Stem Cell Technologies, 1∶100), anti-mouse CD24 APC (Biolegend, 1∶100), anti-mouse CD29 Pacific Blue (BioLegend, 1∶100), anti-mouse CD14 FITC (eBiosiences, 1∶80) and anti-mouse ckit PE (Clone ACK4, Cedarlane Laboratories, 1∶50). Cells were sorted using a BD FACS Aria II Cell Sorter.

### RNA isolation

Mammary glands (#4) were excised from 10–12 week old Balb/c female mice and snap frozen. Three mice were sacrificed for each tissue type and mammary gland developmental time point. RNA was isolated using TRIzol (Invitrogen) and quantified using a NanoDrop (ThermoScientific). RNA was also isolated from MECs and cells lines using TRIzol. RNA integrity was analyzed on a 1% agarose gel prior to cDNA preparation. RNA generated from mature luminal, luminal progenitor, and alveolar progenitor fractions was amplified by the BCM Genomic and RNA Profiling Core using the NuGen WT-Ovation Kit (NuGen).

### RT–PCR

For each sample, 1–3 ug of RNA was DNase treated (Invitrogen, DNase I), primed with either oligo dT or random primers and reverse transcribed using Superscript II (Invitrogen). Alternatively, RNA was DNAse treated using DNA-Free kit (Ambion) and reverse transcribed using the High Capacity RNA to cDNA kit (Applied Biosystems). To analyze splice variants of mPINC, primers designed to the first and last exons of both *mPINC1.0* and *mPINC1.6* were used to amplify cDNA from 12 day pregnant Balb/c mammary glands using Taq polymerase (Invitrogen). The PCR products were run on a 1% low melting point (LMP) agarose gel, excised and cloned into the TOPO TA pCR2.1 vector (Invitrogen) for sequencing. To identify the exonic structure of the new splice variants, sequences were aligned to the *mPINC* gene locus using UCSC Blat and Multalin.

### Quantitative PCR

The ΔΔCT method was used to determine relative levels of gene expression. All primer sets were tested using a 10-fold dilution series containing 5 dilutions and primer efficiencies were measured to determine the validity of using the ΔΔCT method. For SYBR Green qPCR, *mPINC* primers were designed to detect each isoform specifically (*mPINC1.0*-SYBR-f-gctatgtgaaggaacactgcaaag, *mPINC1.0*-SYBR-r-tctcctcttggacagaatccactt, *mPINC1.6*-SYBR-f-gtgaaggaacactgcaaagagct, *mPINC1.6*-SYBR-r-ccccagagtgctccatgttt). Two housekeeping genes were used as normalizers, beta-actin and cyclophilin b (*Actb* F-gcaacgagcggttccg, *Actb* R-cccaagaaggaaggctgga, *Ppib* F-ttgccatccagccactcag, *Ppib* R-tgagcactggggagaaagg). Each biological replicate was run in triplicate using SYBR Green qPCR Master Mix (Applied Biosystems). The ΔΔCT method was used to obtain values of the relative levels of *mPINC1.0* and *mPINC1.6* by using both beta-actin and cyclophilin b as internal reference genes and setting all samples relative to the sample with the lowest amount of detectable target (Ct<35). Samples with Ct values greater than 35 were considered undetectable. For Taqman qPCR, the following probe sets were purchased from Applied Biosystems: *mPINC1.0*-Mm03456232_m1, *mPINC1.6*-Mm03456230_m1, *mPINC*-Mm03456228_m1, *Gapdh*-Mm99999915_g1, *Csn2*-Mm00839664_m1, *Wap*-Mm00839913_m1, *Ltf*-Mm00434787_m1, *Edn1*-Mm00438656_ml, *Wnt4*-Mm01194003_m1, *Dlk2*-Mm01281511_g1, *Ly6a*-Mm00726565_s1, *Pip*-Mm00476800_m1, *Chi3l1*-Mm00801477_m1, *Itgb3*-Mm00443980_m1, *Ednra*-Mm01243722_m1. Each biological replicate was run in triplicate using Taqman Gene Expression Master Mix (Applied Biosystems).

### *In situ* hybridization

Mammary glands were removed from 12 day pregnant Balb/c female mice, fixed for 4 hrs in 4% PFA at 4°C then processed and embedded in paraffin. Antisense and Sense probes were PCR amplified from plasmids containing full length *mPINC1.0* or *mPINC1.6* and gel purified. The primers (*mPINC1.0* antisense F-T7-tcctgcattaacccttcatca, *mPINC1.0* antisense R-tggattctgtccaagaggaga and *mPINC1.6* antisense F-T7-ttgctcacaatcatccctca, *mPINC1.6* antisense R-cacttcctgctcaggtgtca) generated probes that were 167 bases for *mPINC1.0* and 225 bases for *mPINC1.6*. Digoxigenin-labeled probes were *in vitro* transcribed by T7 polymerase using 500 ng of template and DIG-labeling mix (Roche). Following rehydration, sections were treated with Proteinase K (10 ug/ml), post-fixed in 4% PFA for 15 min, acetylated for 10 min and prehybridized for 1 hr in Amresco Hybe Solution (AMR-0973) at 55°C. Antisense and Sense control probes were incubated with sections overnight at 55°C diluted to 0.1 ng/ul. Excess probe was eliminated by treatment with RNase/T1 cocktail (Ambion) in 2× SSC for 30 min followed by stringent washes. Sections were then blocked in TBS with 3% sheep serum and 0.3% triton-X 100 for several hours at room temperature before adding an anti-Digoxigenin-Alkaline Phosphatase antibody (Roche, 1∶250) in block solution with 2% sheep serum overnight. The next day sections were washed and stained using BM Purple (Roche). After stopping the color reaction, sections were rinsed with water and mounted with Vectashield Mounting Medium (Vectorlabs).

### HC11 cell culture

HC11 cells were grown in 6 well plates at 37°C in 5% CO_2_ in RPMI 1640 media (Gibco) supplemented with 10% bovine calf serum (SAFC Biosciences), 2 mM glutamine (Gibco), 5 ug/ml bovine insulin (Sigma), 10 ng/ml murine EGF (Millipore), and 50 ug/ml gentamycin (Sigma). Upon confluency, cells were grown for an additional 3 days and then incubated overnight in priming media (10% stripped donor horse serum (SAFC Biosciences), 2 mM glutamine, 5 ug/ml bovine insulin, 50 ug/ml gentamycin in RPMI 1640 media). Cells were differentiated in priming media with the addition of hormones (1 ug/ml Prolactin (National Hormone and Pituitary Program, NIDDK, National Institutes of Health, Bethesda) and 1 ug/ml Hydrocortisone (Sigma)) for either 24 hrs or 72 hrs before harvesting the cells for RNA isolation. Domes were counted following 2 days of hormone treatment from nine 20× images/experiment.

### Plasmid construction

LeGO lentiviral vectors were kindly provided by Dr. Kristoffer Weber [69]. LeGO-iCer2 vector was cut with BsrGI and NotI to release the IRES-Cerulean, ends were filled with T4 polymerase and the cut vector was religated to create LeGO-IF. *mPINC1.0*, *mPINC1.6* and the deleted conserved region (*DCR*) mutant were amplified from plasmids containing the full length sequences of *mPINC1.0* and *mPINC1.6* (GenBank #s DQ059755 and DQ059756) adding RE sites to clone into the remaining sites of the LeGO-IF MCS. All plasmids were sequence-verified prior to lentiviral production.

### Lentiviral production and titering

Lentiviral vectors (LeGO-GFP, LeGO-1.0, LeGO-1.6 or LeGO-DCR) were co-transfected with packaging vectors (pCMV-VSVG, pMDLg/pRRE, pRSV-Rev) into 293-T cells using Fugene 6 (Roche). Viral supernatant was collected at 48 and 72 hrs post-transfection, pooled and filtered through 0.45 uM filters to remove cellular debris. Filtered viral supernatant was concentrated using the Beckman Coulter Optima ultracentrifuge (SW32Ti rotor) at 25,000 rpm for 1 hr and 45 min. Ultracentrifuged virus was resuspended in DMEM. LENTI-X Elisa kits (Clontech) were used to titer the virus using LeGO-GFP as a calibrator. The LeGO-GFP virus was titered first by methods previously described based on FACs analysis of GFP expression in transduced 293-T cells. HC11 cells were transduced with lentivirus at a MOI of 20. Cells were expanded following two passages, aliquoted and frozen for experiments.

### Transfection of siRNAs

HC11 cells were transfected with siRNAs (10 nM/well) at 60–80% confluency in 6 well plates using Lipofectamine RNAiMAX reagent (Invitrogen). Silencer Select siRNAs (Ambion) were used to target *mPINC1.0* (n273107), *mPINC1.6* (n254312) and *DCR2* (n254314). The controls included mock tranfection without siRNAs and a siNEG, Silencer Select Negative Control #1 (4390843).

### RNA immunoprecipitation (RIP) assay

RNA immunoprecipitation (RIP) assays were carried out following published protocols with some modifications [13], [23]. Cells from 150 mm plate were harvested by trypsinization and re-suspended in 2 ml PBS, 2 ml nuclear isolation buffer (1.28 M sucrose; 40 mM Tris-HCl pH 7.5; 20 mM MgCl_2_; 4% Triton X-100) and 6 ml water on ice for 20 min. Nuclei were pelleted by centrifugation at 2,500 rpm for 15 min, resuspended in 1 ml of RIP buffer (150 mM KCl; 25 mM Tris pH 7.4; 5 mM EDTA; 0.5 mM DTT; 0.5% NP40; 25 µg/ml leupeptin; 1 mM benzamidine, 10 µg/ml trypsin inhibitor, 25 µg/ml aprotinin, 1 mM PMSF; 100 U/ml SUPERase-in (Ambion)) and mechanically sheared using 25 G needle with 6 strokes. Nuclear membrane and debris were pelleted by centrifugation at 14,000 rpm for 10 min. All lysates were pooled together and aliquoted at 10^7^ cells/IP(about 500 µl of lysate). DNA was degraded with Turbo DNase (Ambion) for 20 min at 37°C followed by adding EDTA to a final concentration of 20 mM and insoluble material was removed by centrifugation for 10 min at 14,000 rpm. Supernatants were pre-cleared by adding 15 µl of rabbit IgG and 20 µl of magnetic A/G beads (Invitrogen) following incubation at 4°C for 2 hrs with gentle rotation. Antibody to EZH2, SUZ12, RbAp46 (Abcam) or MLL1 (Bethyl) were added to pre-cleared supernatants (5 µg each) together with 20 µl of A/G magnetic beads and incubated overnight at 4°C with gentle rotation. The beads were then washed 3 times with RIP buffer followed by one wash in PBS and resuspended in 1 ml of TRIzol-LS (Invitrogen). Co-precipitated RNAs were treated with DNase (Ambion) and reverse transcribed using RNA-to-cDNA kit (Applied Biosystems).

### Gene expression profiling

Microarray analysis was performed on biological duplicates for undifferentiated mPINC knockdown HC11 cells and on biological triplicates for differentiated mPINC knockdown and overexpression HC11 cells. RNA was isolated using Trizol, followed by purification using RNeasey MinElute Cleanup Kit (QIAGEN). The BCM Genomic and RNA Profiling Core conducted sample quality checks using the Nanodrop ND-1000 and Agilent Bioanalyzer Nano chip. 50 ng of total RNA was amplified and Cy3-labeled using the Agilent Quick Amp Labeling Kit (for one-color) Protocol Version 6.5. Hybridization to Agilent Sure Print 3 Mouse GE 8×60K Microarrays was performed at the BCM Genomic and RNA Profiling Core. Array data were quantile normalized, after which significantly regulated genes were identified by comparing control with groups using t-test (log-transformed data, two-sided) and fold change (ratio of averages of the two groups). Java TreeView [70] represented expression patterns as color maps, where expression values were centered on the average of the control group. Microarray data are available on the Gene Expression Omnibus (GSE38052). Gene ontology (GO) analysis was carried out using DAVID [71], [72].

### RNA structure prediction

The spliced, full length transcripts of interest were submitted to RNA secondary structure analysis via RNAstructure version 5.3 [73]. Folding was performed using the partition function algorithm, which enables the prediction of putative RNA pseudoknots with ProbKnot [74]. We restricted the size of helical regions in the final display to ≥4 base pairs and exported circle plots annotated with base-pair probabilities from the partition function. The 3D modeling of common, highly probable secondary structure interactions was performed with the MC-FOLD/MC-SYM pipeline [75]. MC-FOLD predictions were restricted to the topologies predicted by RNAstructure, while the resulting non-canonical secondary structure with the highest score was submitted to MC-SYM. Evolutionary conservation of RNA structure was performed using SISSIz in a sliding-window framework [44]. The 46-way Multiz alignments associated to the *PINC* locus were downloaded from the UCSC genome browser (hg19). Alignment blocks were then merged together with an ad-hoc perl script, realigned with Clustalw2 [76], broken into windows of 200 columns that overlap by 50, and removed identical sequence and sequences with more than 75% gaps. We qualify hits presenting a Z-score below −3 (P-value≤1.35×10E-3) as bearing evolutionary conserved structures.

### Statistics

One-way ANOVA, followed by a Tukey post-test, was performed on all experiments involving 3 or more groups and the graphs in each figure show significance using asterisks to denote the p-values (***p<0.001, **p<0.01, *p<0.05). For experiments with only 2 groups, unpaired Student *t*-tests were performed.

## Supporting Information

Figure S1 *mPINC* is expressed at varying levels in alveolar cells of the midpregnant gland. (A) In situ hybridization shows that some alveolar cells have higher levels of *mPINC1.0* (large arrow) while some alveolar cells appear to express less (small arrow) (10×). (B) *mPINC1.6* is also expressed at higher levels in some alveolar clusters (large arrow) than in others (small arrow) (20×).
(TIF)

Table S1 436 gene probes are significantly changed in *mPINC* knockdown cells. This list includes all genes that are significantly altered following *mPINC* knockdown (p<0.01, FC>1.8 for either siPINC1.0/1.6 or siPINC relative to siNEG).
(XLSX)

Table S2 GO analysis of genes altered by *mPINC* knockdown reveals GO terms associated with development and differentiation. This spread sheet includes lists of genes identified by microarray analysis that are associated with each GO term (adjusted p value>0.05) by DAVID GO analysis.
(XLSX)

Table S3 181 genes are oppositely regulated by *mPINC* knockdown and overexpression. This list includes all genes that are significantly altered in the opposite direction between *mPINC* knockdown (undifferentiated or differentiated) and *mPINC* overexpression (p>0.01, FC>1.4).
(XLSX)
