# Supplementary figures and images for: Pregnancy-Induced Noncoding RNA (PINC) Associates with Polycomb Repressive Complex 2 and Regulates Mammary Epithelial Differentiation
Source: PLoS Genet. 2012 Jul 26;8(7):e1002840. doi: 10.1371/journal.pgen.1002840 (PMC3406180; doi:10.1371/journal.pgen.1002840)

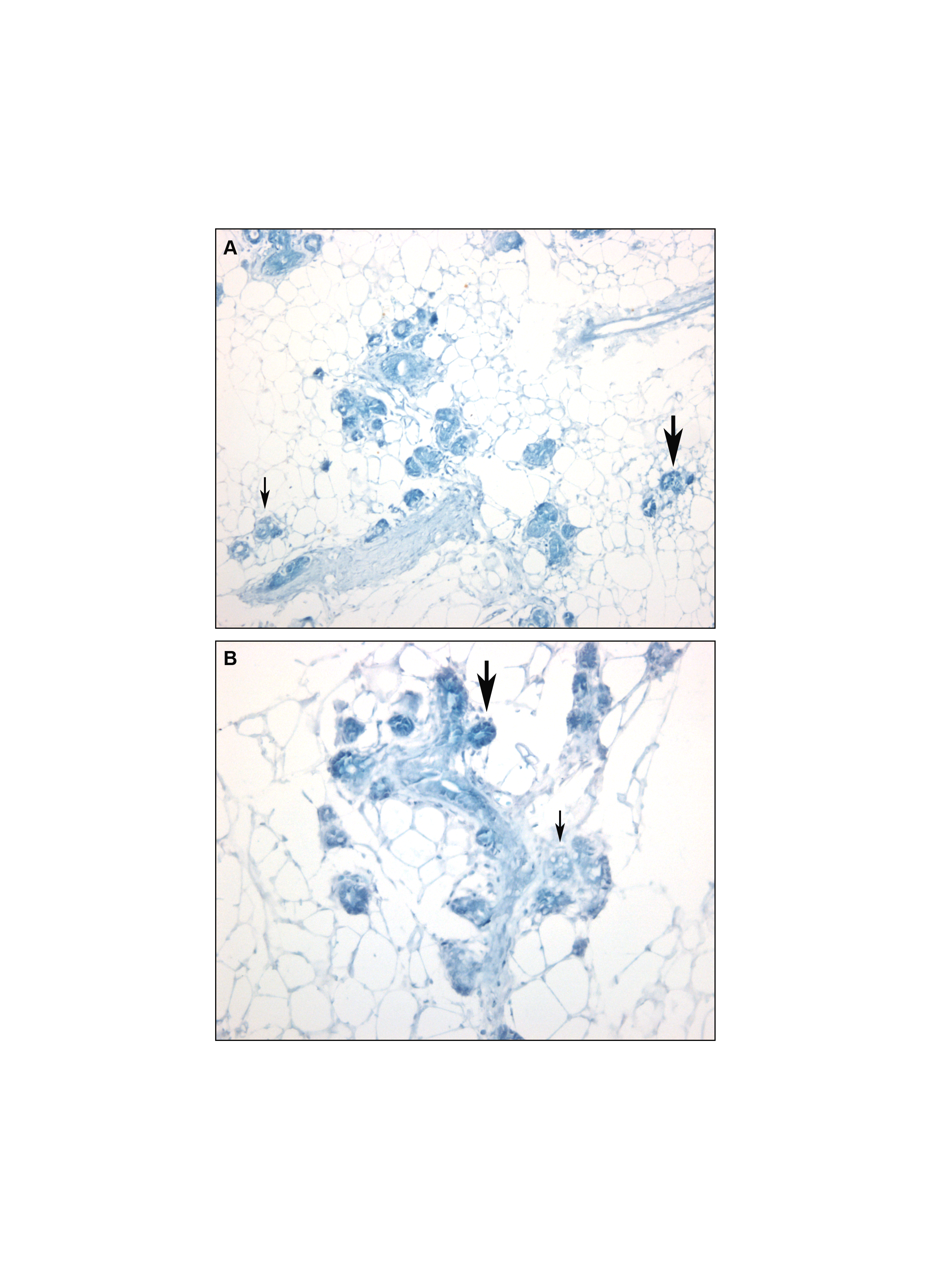

Supplement: Figure S1 — mPINC is expressed at varying levels in alveolar cells of the midpregnant gland. (A) In situ hybridization shows that some alveolar cells have higher levels of mPINC1.0 (large arrow) while some alveolar cells appear to express less (small arrow) (10×). (B) mPINC1.6 is also expressed at higher levels in some alveolar clusters (large arrow) than in others (small arrow) (20×). (TIF) [file pgen.1002840.s001.tif]
